# Cryo-EM study and *in vivo* chemical mapping of the *Methanosarcina acetivorans* ribosome and its dimerization *via* a repurposed enzyme and translation factor

**DOI:** 10.1016/j.jbc.2025.110686

**Published:** 2025-09-04

**Authors:** George N.R. Fordjour, Anwesha Ghosh, James G. Ferry, Jean-Paul Armache, Philip C. Bevilacqua, Katsuhiko S. Murakami

**Affiliations:** 1 Department of Biochemistry and Molecular Biology, Penn State University, University Park, Pennsylvania, USA; 2 Center for Structural Biology, Penn State University, University Park, Pennsylvania, USA; 3 Center for RNA Molecular Biology, Penn State University, University Park, Pennsylvania, USA; 4 Center for Eukaryotic Gene Regulation, Penn State University, University Park, Pennsylvania, USA; 5 Molecular Machines Mechanism and Structure Predoctoral Training Program, Penn State University, University Park, Pennsylvania, USA; 6 Department of Chemistry, Pennsylvania State University, University Park, Pennsylvania, USA

**Keywords:** ribosome, cryo-electron microscopy, archaea, methanogen, RNA chemical structure probing, structural biology

## Abstract

Despite the overall conservation of ribosomes across all domains of life, differences in their 3D architecture, rRNA sequences, ribosomal protein composition, and translation factor requirements reflect lineage-specific adaptations to environmental niches. In the domain *Archaea*, structural studies have primarily focused on nonmethanogenic thermophiles and halophiles, leaving it unclear whether these represent the broader *Archaea* domain. Here, we report the cryo-electron microscopy (cryo-EM) structure of the ribosome from *Methanosarcina acetivorans*, a previously unreported high-resolution structure from a model mesophilic methanogenic archaeon. Compared to ribosomes from extremophiles, the *M. acetivorans* ribosome has a simplified architecture, lacking paralogous duplications and containing a reduced complement of ribosomal proteins. Structures of the large subunit (50S) from cells grown with either methanol or acetate show conserved rRNA folding and protein composition. High-resolution structures of the 50S subunit from the two growth substrates enabled us to investigate structural properties that may influence *in vivo* dimethyl sulfate reactivity, an orthogonal chemical approach used to probe RNA structure. We observed good agreement between *in vivo* dimethyl sulfate reactivity and ribosome structure. Finally, we identify a previously uncharacterized ribosome dimerization mode involving only 50S subunits and mediated by a heterotetrameric complex of PurH and aEF2-proteins with alternative metabolic and translational roles. This macromolecular assembly, which we term the methanogen ribosome dimerization factor, likely mediates ribosome hibernation, revealing an alternative regulatory mechanism in translation.

Ribosomes are essential macromolecular machines responsible for protein synthesis. Unlike DNA and RNA polymerases, similar enzymes key to the central dogma, which are found in both viruses and living organisms, ribosomes are found exclusively in metabolizing cells. This makes ribosome the defining element that distinguishes living organisms from nonliving biological entities. The ribosome core, consisting of ribosomal RNAs (rRNAs), is highly conserved across the three domains of life. However, there are some notable differences in the structure, sequence, and composition of rRNA and ribosomal proteins (r-proteins), and translation factors functioning at the stages of initiation, elongation, and termination reveal their unique evolutionary diversity and reflect their adaptation to environmental factors (1, 2, 3). This can lead to differences in ribosome structures between organisms and even heterogeneity in the structures of ribosomes in the same organism (4).

Despite their classification as prokaryotes, the domain *Archaea* contains ribosomes that closely resemble those in the domain *Eukarya*. Most r-proteins and ribosome biogenesis factors are conserved between *Archaea* and *Eukarya* domains. Similarity and simplified archaeal ribosome architecture and biogenesis make species from the domain Archaea good model systems for elucidating the structure and function of protein translation in the *Archaea*-*Eukarya* lineage (5, 6).

Structural studies on archaeal ribosomes to date have been focused almost exclusively on nonmethanogenic thermophiles (7, 8, 9, 10, 11, 12, 13, 14) or halophiles (11, 15). Such extremophiles have developed molecular adaptations, such as having proteins with prominent hydrophobic core and increased salt-bridge networks of proteins for enhancing their thermal stability in thermophiles, and increasing negative surface charge due to high acidic amino acid content, which compensates for the extreme ionic conditions in halophiles (16). However, the extent to which ribosomes from nonmethanogenic thermophiles and halophiles reflect general features of the entire *Archaea* domain, particularly as compared to nonmethanogens and nonextremophiles, remains an open question.

Given their abilities to adapt to versatile environments, there are also questions around how the archaea regulate their translational machinery, especially since their ribosome structures share characteristics with both Bacteria and Eukarya and can become heterogeneous in response to abiotic and biotic factors such as environmental stressors, energy sources, and viral infections (5, 16, 17, 18, 19). However, there are limited examples of how differences in ribosome architecture may influence translation in the domain *Archaea*.

*Methanosarcina acetivorans* is a methanogenic mesophilic (∼35 °C), nonhalotolerant (0.4 M NaCl), neutrophile (pH∼7) and strictly anaerobic archaeon that has emerged as a model organism for its methane production and scavenging (20). Originally isolated from marine sediments, *M. acetivorans* has the largest known genome among the domain Archaea (21), which underlies its remarkable metabolic versatility, enabling the utilization of diverse carbon and energy substrates, including acetate, methanol, and methylamines (18, 20). Of these substrates, most biochemical studies have been conducted comparing methanol to acetate growth conditions (22, 23, 24, 25, 26), and have shown distinct gene expression profiles and physiological states for the two substrates. Notably, in the past few years, there has been increasing interest in RNA structure and function relationships in this organism (23, 27). Recent *in vivo* RNA structure probing using dimethyl sulfate (DMS) revealed growth substrate-dependent differences in rRNA accessibility, suggesting potential structural or compositional variation in the ribosome in response to the carbon and energy source in *M. acetivorans* (27), which prompted us to investigate changes in the structure of the ribosome with these different growth substrates.

In the domain Bacteria, such as *Escherichia coli*, ribosome hibernation is an essential strategy for survival during nutrient deprivation or stress routinely encountered in the environment. Under these conditions, 70S ribosomes including the large (50S) and small (30S) subunits are converted into translationally inactive 100S dimers through the coordinated action of ribosome hibernation factors, primarily the ribosome modulation factor (28) and the two additional hibernation factors: HPF and RaiA (29). The resulting ribosome dimers are inactive but stable, allowing the cell to preserve ribosomes for future reactivation during favorable growth conditions. Eukaryotic cells also employ ribosome hibernation mechanisms, which are increasingly recognized as conserved and physiologically important across species. Importantly, ribosome hibernation in eukaryotes is not limited to stress response but also plays critical roles in oogenesis, egg maturation, and early embryonic translation control (30). Compared to studies of ribosome hibernation in Bacteria and Eukarya, structural and functional studies of ribosome hibernation in *Archaea* are still in the early stages (31).

In this study, we determined the cryo-electron microscopy (cryo-EM) structure of the *M. acetivorans* 70S ribosome from cells grown with either methanol or acetate as substrates to provide insight into how distinct energy sources may influence protein production *via* ribosomes, as well as evolution of ribosomes in *Archaea*-*Eukarya* lineage. A portion of this study focuses on relating our recent rRNA chemical structure probing (27) to the high-resolution ribosome structures solved herein, which support the *in vivo* relevance of our structures. Although characterizing the ribosome structure, we also identified a novel dimerization of the 50S subunit of ribosome, mediated by a previously undefined molecular assembly that we term the methanogen ribosome dimerization factor (MRDF). Unlike known ribosome dimerization factors, MRDF is composed of proteins with established functions, *de novo* purine biosynthesis and GTP-binding translation factor, suggesting a ribosome hibernation by using these factors as sensors for stress and for starvation for nutrients encountered in the environment.

## Results

### Cryo-EM structure determination of the *M. acetivorans* ribosome from methanol-grown cells

**Figure 1:**
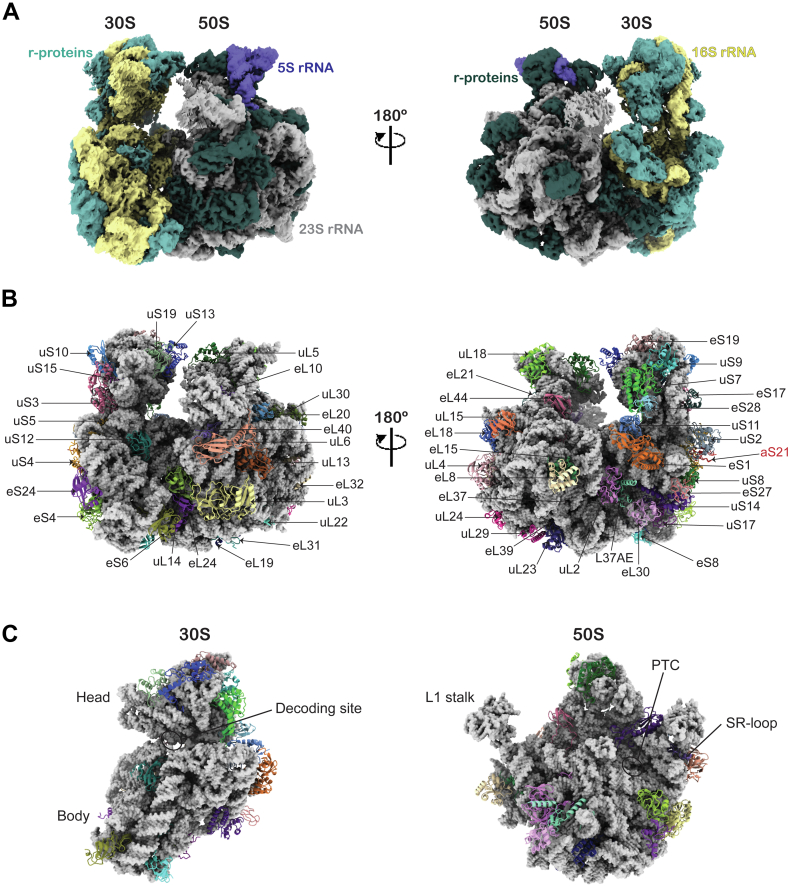
**Cryo-EM structure of the***Methanosarcina****acetivorans* ribosome**. *A*, cryo-EM map of the *M. acetivorans* 70S ribosome. rRNAs (23S, 5S, and 16S) and r-proteins are indicated. *B*, structure of the *M. acetivorans* 70S ribosome showing r-proteins. r-proteins are labeled. aS21 represents the small putative zinc finger domain-containing protein (UniProt: Q8TQR8), highlighted in *red*. *A*–*B*, are of similar orientation. (“u” = universal, “e” = shared by archaea and eukaryotes, “S” = small subunit, and “L” = large subunit) (78). *C*, the interface between 50S and 30S subunits. Several key functional sites involved in protein translation are indicated. The 30S and 50S subunits are oriented 45° clockwise and counterclockwise, respectively, relative to the *right panel* of (*B*).

We purified ribosomes from *M. acetivorans* grown with either methanol or acetate as growth substrates. *M. acetivorans* grown with methanol yielded more ribosomes than those grown with acetate due to producing a higher cell density (32). During cryo-EM data processing, ribosome particles were found as 70S, containing 50S + 30S subunits, or 50S subunit alone. Optimal yield of 70S ribosome particles from methanol-grown cells enabled cryo-EM density map reconstruction at a resolution of 2.92 Å (Figs. 1, S1, Table S1, and Movie S1), allowing detailed analysis of RNA posttranscription modifications, as well as the secondary and tertiary structures of ribosomal RNAs, the composition of r-proteins and their interactions. Although the ribosomes were isolated using a simple sucrose cushion method, we did not observe any cryo-EM density in the 70S ribosome corresponding to tRNA or mRNA in the intersubunit space between the 50S and 30S subunits.

We identified 24 and 30 annotated r-proteins in the 30S and 50S subunits of the *M. acetivorans* ribosome, respectively, and a putative zinc finger domain-containing protein aS21 (Fig. 1*B* and Movie S2). The 30S subunit showed modest resolution, ranging 3.0 Å to 6.5 Å, due to its inherent mobility relative to 50S subunit (Fig. S2*A*). To improve the quality of the 30S, we used particle subtraction to computationally remove the 50S subunit, followed by local 3D refinement of the 30S subunit. This approach improved the overall resolution of 30S subunit to 3.11 Å (Fig. S3).

### RNA modifications and structural differences of ribosomes isolated from methanol- and acetate-grown cells

**Figure 2:**
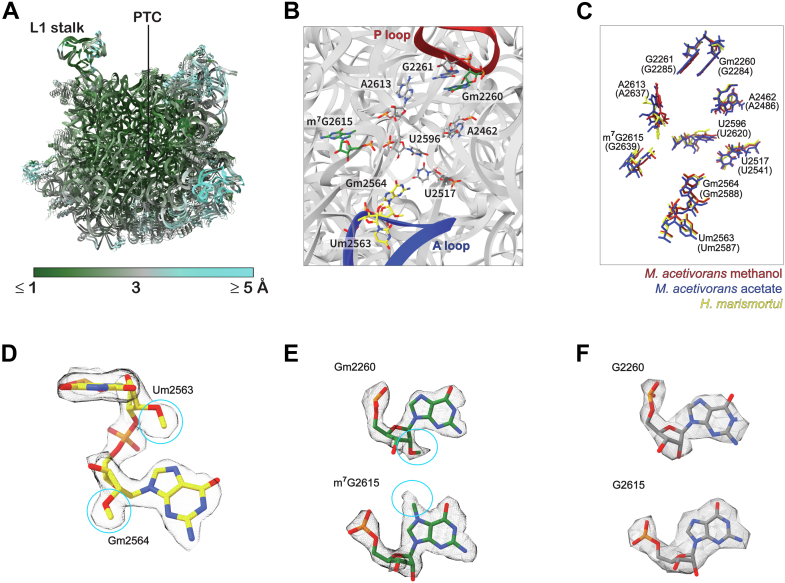
**Comparison of the 50S subunits of the *Methanosarcinaacetivorans* ribosome grown with either methanol or acetate substrate**. *A*, R.M.S.D of 50S subunits of ribosome isolated from methanol- and acetate-substrates grown cells. PTC and L1-stak are indicated. *B*, close-up view of PTC of *M. acetivorans* 50S subunit shows modified rRNA isolated from methanol-substrate grown cells only (*green*), from both methanol- and acetate-substrates grown cells (*yellow*) and canonical nucleotides essential for ribosome activity (*gray*). Nucleoside positions are labeled according to *M. acetivorans* 23S rRNA numbering. *C*, superposition of the PTC of *M. acetivorans* 50S subunit of ribosome isolated from methanol-substate grown cells (*red*), acetate-substrate grown cells (*blue*), and from 50S subunit of *Haloarcula marismortui* (PDB:4V9F) (*yellow*). Nucleoside positions are labeled as in (*B*) and for *H. marismortui* in parentheses *D*–*E*, cryo-EM maps (*white meshes*) for representative modified nucleosides identified in (*D*) both methanol- and acetate-substrate grown *M. acetivorans* cells (Gm2564 and Um2563) and (*E*) only in methanol-substate grown cells (Gm2260 and m^7^G2615). Density maps corresponding to posttranscriptional modifications are indicated by *cyan circles*. *F*, canonical nucleotides at G2260 and G2615 in acetate-substate grown cells. PDB, Protein Data Bank; PTC, peptidyl transferase center.

To investigate the rRNA modification of ribosomes isolated from methanol- and acetate-grown cells, particularly around the peptidyl transferase center (PTC), we increased nominal resolutions of the 50S subunit ribosomes by combining particles containing 50S subunit (either 50S subunit alone or 50S within the 70S form) and obtained resolutions at 2.38 Å from methanol-grown cells (Fig. S1) and at 2.85 Å resolution from acetate-grown cells (Fig. S4). In both cryo-EM maps, local resolution around the PTC reached approximately 2.5 Å or higher (Fig. S2), allowing us to identify coordinated metals without ambiguity including magnesium, potassium, and zinc (Fig. S5). Metal ion assignment was validated by comparison previously solved high-resolution ribosome structures and by analyzing the number and geometry of coordinating groups (7, 9, 12, 33). Our 50S subunits from methanol- and acetate-grown cells were similar in overall structure, rRNA secondary and tertiary structures and r-protein composition (Figs. 2*A* and S6*A*). Structural alignment yielded root-mean-square deviations (R.M.SD) below 3.0 Å for most rRNA regions, with slightly elevated values localized to flexible regions, as indicated by higher B-factor distribution (Figs. 2*A* and S6, *B* and *C*).

Only a modest difference in RNA modification profiles were observed between the ribosomes isolated from methanol-grown and acetate-grown cells. Compared to those from acetate grown cells (Fig. 2*B*), 50S ribosomal subunits from methanol-grown cells do carry two additional modifications (Fig. 2*B*): G2260 has a 2′-O-methylation (Gm2260) and G2615 has an N7-methylation (m^7^G2615), which is positioned near the PTC (Fig. 2, *E* and *F*). Notably, the 2′-O-methylation has been identified in the PTC in domain *Bacteria* as well. In *E. coli*, the 2′-O-methylation occurs at G2251 (34), which is at the equivalent position as G2260/G2265 in *M. acetivorans*, where “G2260” numbering is from our cryo-EM structure and “G2265” numbering is from that used in our previous *in vivo* RNA probing study (27). Two other modifications are found, at U2563 (2′-O-methylation, Um2563) and G2564 (2′-O-methylation, Gm2564) of ribosomes from cells grown with both substrates (Fig. 2*D*). The modifications observed under both growth conditions are matched with those identified in the *Haloarcula marismortui* ribosome (Protein Data Bank (PDB): 4V9F) (Fig. 2*C*) (12). Superposition of the *M. acetivorans* 50S subunit with the *H. marismortui* reference structure revealed close alignment of most residues at the PTC, although subtle differences were detected at A2613 and U2596, which are the two most flexible residues in the PTC (35, 36) and at Gm2564, which contacts the 3′-terminal CCA end of the A-site tRNA (Fig. 2*D*) (37). A previously reported N4-acetylcytidine rRNA modification in the *Thermococcus kodakarensis* ribosome (7) was not detected in the *M. acetivorans* ribosome.

### Structural comparison of ribosomes between *M. acetivorans* and thermophiles

**Figure 3:**
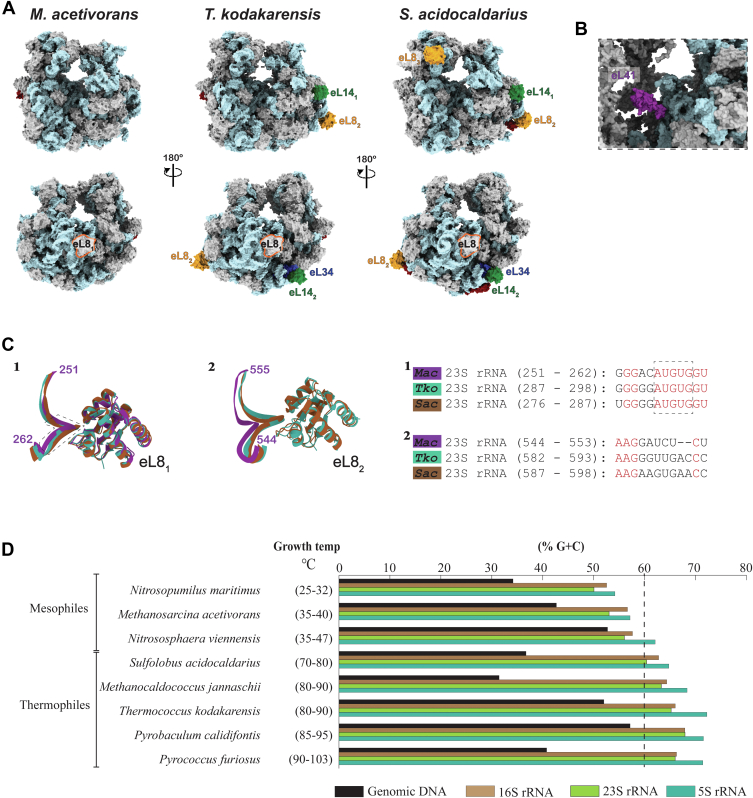
**Comparison of the ribosomes from mesophiles and thermophiles**. *A*–*B*, comparison of ribosomal protein (r-protein) composition in *Methanosarcina acetivorans* (*left*), *Thermococcus kodakarensis* (PDB: 6SKF, *middle*), and *Sulfolobus acidocaldarius* (PDB: 8HKY, *right*) ribosomes. Conserved r-proteins present in all three species are shown in *gray*, while additional r-proteins unique to *T. kodakarensis* and *S. acidocaldarius* are highlighted and labeled. eL8_1_, present in all ribosomes, is also depicted. Duplicated orthologs are denoted with subscripts. *C*, interactions between 23S rRNA and eL8-related proteins*. Left*: interactions between rRNA and eL8_1_ observed in all three ribosomes. *Middle*: interactions between rRNA and eL8_2_, found only in *T. kodakarensis* and *S. acidocaldarius* ribosomes. *Right:* nucleotide sequences at the eL8 interaction sites; conserved bases are highlighted in *red*. Region 2 shows a loosely conserved RNA motif, with *M. acetivorans* 23S rRNA featuring a deletion in this region. *D*, comparison of G + C content in whole-genome DNA and rRNA genes from mesophilic and thermophilic archaea. Growth temperatures are indicated in parentheses. PDB, Protein Data Bank.

Structural studies of archaeal ribosomes have been focused on nonmethanogenic thermophilic species, which often exhibit adaptations to extreme temperatures. To find adaptations for ribosome thermostability, we compared the cryo-EM structures of the mesophilic *M. acetivorans* ribosome (this study) with those of previously reported ribosomes isolated from the thermophilic anaerobic euryarchaeota *T*. *kodakarensis* (PDB: 6SKF) (7) and from thermophilic aerobic crenarchaeota *Sulfolobus acidocaldarius* (PDB: 8HKY) (9). *M. acetivorans* ribosome has a simplified r-protein composition (Fig. 3*A* and Table S2). For example, *T. kodakarensis* and *S. acidocaldarius* ribosomes contain two (eL8_1_ and eL8_2_) and three (eL8_1_, eL8_2,_ and eL8_3_) paralogues of ribosomal protein eL8. These proteins bind kink-turn motifs found in the 23S (for eL8_1_ and eL8_2_) and 16S rRNAs (for eL8_3_), respectively. In contrast, the *M. acetivorans* ribosome contains only a single eL8, which binds to a conserved AUGUG motif in the 23S rRNA (Fig. 3*C*). Notably, the *M. acetivorans* 23S rRNA is two bases shorter in the kink-turn motif of the second eL8 binding site found in the *T. kodakarensis* and *S. acidocaldarius* ribosomes, likely explaining its absence in the *M. acetivorans* ribosome. In addition, *M. acetivorans* ribosome lacks genes encoding several r-proteins such as eL14, eL34, and eL41 that are present in the thermophilic archaea (Table S2). Except for eL41, which is located at the interface between the 50S and 30S subunits (Fig. 3*B*), all r-proteins unique in thermopiles are located on the ribosome periphery, away from the catalytic core, suggesting that they primarily function in the ribosome thermostability.

We also compared the G + C content of whole genomic DNAs and ribosomal RNAs among *M. acetivorans*, *T. kodakarensis*, *S. acidocaldarius*, and other archaeal species (Fig. 3*D* and Table S3). Although the G + C content of genomic DNA showed no consistent relationship with their optimal growth temperatures (G + C contents: *M. acetivorans*: 43%; *T. kodakarensis*: 52%; *S. acidocaldarius*: 37%), the rRNA G + C content strongly correlated with their optimal growth temperatures (G + C contents: *M. acetivorans*: 53%; *T. kodakarensis*: 65%; *S. acidocaldarius*: 60% in 23S rRNA and optimal growth temperatures of 35−40 °C, 80−90 °C, and 70−80 °C, respectively). rRNAs from mesophilic archaea, including *M. acetivorans*, have lower G + C content than their thermophilic counterparts (Fig. 3*D* and Table S3), suggesting higher G + C content contributing to thermal stabilization of rRNA structure in thermophiles, consistent with a previous study focusing on tRNA (38).

### Dependence of *in vivo* DMS chemical reactivity on SASA and number of hydrogen bonds

We previously reported reactivity of the chemical DMS, which methylates As and Cs on the Watson-Crick-Franklin (WCF) face at specific positions in rRNA that was isolated from methanol- and acetate-grown *M. acetivorans* (27). In that study, DMS reactivity was mapped onto the secondary structures of the rRNA determined by comparative analysis, and reasonable agreement was found, in that DMS reactivity mapped largely to unpaired nucleotides. However, we had no 3D structures of the ribosomes and so were unable to judge any influences on the reactivity beyond base pairing status. We thus begin here by considering the DMS reactivity of the rRNA from *M. acetivorans* grown with methanol or acetate according to the structures we solved herein.

Given that we have high-resolution *M. acetivorans* 50S ribosome structures from two different growth substrates, we wanted to connect our previously published DMS data, which captures *in vivo* information, with our structures. Although the reactivities of the bases with DMS are indicators of base pairing status, they can be influenced by other factors. For instance, DMS reactivity can be affected by RNA-protein interactions and surrounding RNA structure, as well as dynamic properties of the nucleotides (27, 39, 40, 41). As a proxy to these factors, we used our cryo-EM structures to measure the influence of solvent accessible surface area (SASA) and number of hydrogen bonds on DMS reactivity, both of which have been reported to influence DMS reactivity in RNA (41, 42, 43).

Reactivity *versus* SASA (Å^2^) was plotted for the DMS-reactive atoms A(N1) and C(N3) for every A and C in 23S rRNA and 5S rRNA under both methanol and acetate growth conditions. Moderate positive correlations between SASA and DMS reactivity were found for methanol and acetate growth conditions, with r values of 0.44 and 0.39, respectively (Fig. S7, *A* and *B*), indicating that solvent accessible bases tend to have enhanced DMS reactivity. To look for any trends of differential DMS reactivity on SASA, we plotted change in DMS reactivity *versus* change in SASA, where “change” is defined as methanol minus acetate growth conditions. However, no correlation was found, with an r value of −0.06 for an attempted linear fit (Fig. S7*C*). Indeed, most residues have the same SASA value between growth substrates, putting them on or near the y-axis (Fig. S7*C*), consistent with overall similarity of the structures in the two conditions described above (Fig. 2*A*).

Next, we plotted DMS reactivity *versus* number of hydrogen bonding interactions present under each of the growth substrates for every A and C in 23S rRNA and 5S rRNA. We counted the number of hydrogen bonding interactions (44) to any atom on the nucleotide (base, sugar, and phosphate) of interest. Weak negative correlations were found for both substrates, with r values for methanol and acetate growth conditions of −0.20 and −0.17, respectively (Fig. S8, *A* and *B*). The trend in the data suggests that bases with fewer hydrogen bonds tend to be more DMS reactive, perhaps because they are more dynamic, a property that has been associated with greater chemical reactivity of another chemical probe, SHAPE (45). It is also notable that the maximal DMS reactivity in each column of hydrogen bonding interactions also trends negatively with number of hydrogen bonds, suggesting a decreasing cap for maximal reactivity with number of hydrogen bonds. Plots of change in DMS reactivity *versus* change in number of hydrogen bonding interactions again proved to have no correlation. Indeed, most residues have the same number of hydrogen bonding interactions (between −1 and +1 difference), putting them on or near the y-axis (Fig. S8*C*), again consistent with overall similarity of the structures in the two conditions.

### Structural basis for differential *in vivo* DMS chemical reactivity

For both growth substrates, the analyses in the previous section revealed a moderate positive dependence of *in vivo* DMS reactivity on SASA and a weak negative dependence of *in vivo* DMS reactivity on number of hydrogen bonds, but it did not uncover a dependence of differential DMS reactivity on either of these factors. To understand any structural basis for differential DMS chemical reactivity, we further investigated the structures of the ribosome solved from these two growth substrates. To identify any trends, we focused on those bases that had the greatest difference in DMS reactivity between the two growth substrates. We previously reported 36 bases that had an absolute DMS reactivity difference greater than 0.5 in 23S rRNA (27). Two of these bases, A976 and A978, were not considered due to insufficient modeling. We first considered the influence of proteins on differential DMS reactivity.

**Figure 4:**
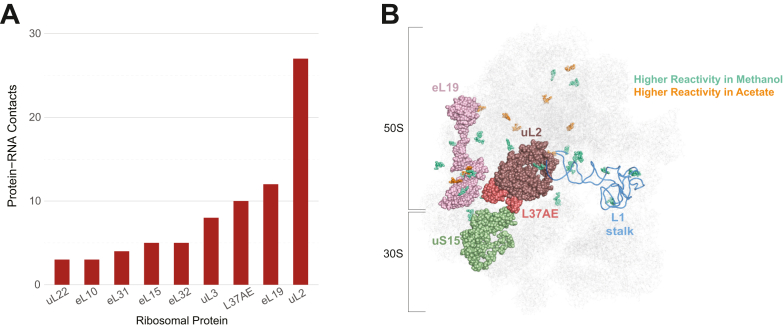
**Protein-RNA contacts for the differentially DMS-reactive nucleotides.***A*, average number of RNA contacts for 50S proteins that are ≤ 4.0 Å from differentially reacting nucleotides. *B*, the 34 differentially DMS-reactive nucleotides from 23S rRNA mapped on the 70S ribosome. *Teal* nucleotides are higher reacting in methanol and *orange* nucleotides are higher reacting in acetate. Proteins with 10 or more RNA contacts that are ≤ 4.0 Å from the differentially reacting nucleotides are shown: L37AE, eL19, and uL2. Protein uS15 is also shown as it is known to interact with uL2 to form an intersubunit bridge between the 50S and 30S. The L1 stalk of 23S rRNA is provided as it too interacts with uL2. DMS, dimethyl sulfate.

We plotted the number of protein-RNA contacts, defined as atoms of a nucleotide within 4 Å of atoms of an r-protein, for the proteins in 50S. Of these, nine r-proteins had contact with the selected bases (Fig. 4*A*). Numbers of protein-RNA contacts found in methanol and acetate were similar and thus averaged, which is also justified because the ribosomal protein occupancy in methanol and acetate was similar. The number of protein-RNA contacts for a given 50S protein ranged from as few as three to as many as 27. The three proteins with the most RNA contacts were L37AE (10 contacts), eL19 (12 contacts), and uL2 (27 contacts), which are highlighted on the 70S structure from methanol (Fig. 4*B*). All three proteins are found near the 50S/30S interface, and many of the differentially reacting nucleotides, colored in teal and orange for more reactive in methanol and acetate, respectively, are localized within 4 Å of these proteins (Fig. 4*B*). Proteins L37AE, eL19, and uL2 coordinate the 30S, forming the intersubunit bridge. Indeed, L37AE also has contacts with 30S proteins and 16S rRNA in the halophilic archaeon, *H. marismortui*, and it forms an intersubunit bridge in yeast (46, 47). Furthermore, uL2, interacts with the highly dynamic L1 stalk of 23S rRNA, which is known to coordinate with tRNAs during translation (48), and with the 30S protein uS15 (Fig. 4*B*). Finally, in addition to its role in being one of the intersubunit bridging proteins, eL19 affects GTPase activation in ribosome aminoacyl tRNA-EF-Tu•GTP complexes critical for translational accuracy (49).

Given the proximity of intersubunit proteins having extensive rRNA contacts to differentially reactivity nucleotides, we inferred that some of the differential reactivity may be due to dynamics and dissociation at the 50S/30S interface (46, 47, 49). Indeed, the 50S and 30S are known to dissociate from one another after translation (50). Figure 4*B* reveals more teal (*i.e.* methanol-reactive) nucleotides near the above three proteins and within the dynamic L1 stalk of 23S rRNA, which might be due to more dissociated ribosomal subunits in methanol. This observation is consistent with the known property of methanol promoting faster growth rates and therefore higher translational levels (20, 21, 22, 46).

We searched for a more detailed molecular mechanism for the differential DMS reactivity by considering several structural factors including differential SASA, differential number of hydrogen bonds, differential protein chain contacts, and RMSD in this region (Table S4). However, no simple trends could be discerned from these analyses.

### Assessing the *in vivo* relevance of the ribosome structures through ROC curves

**Figure 5:**
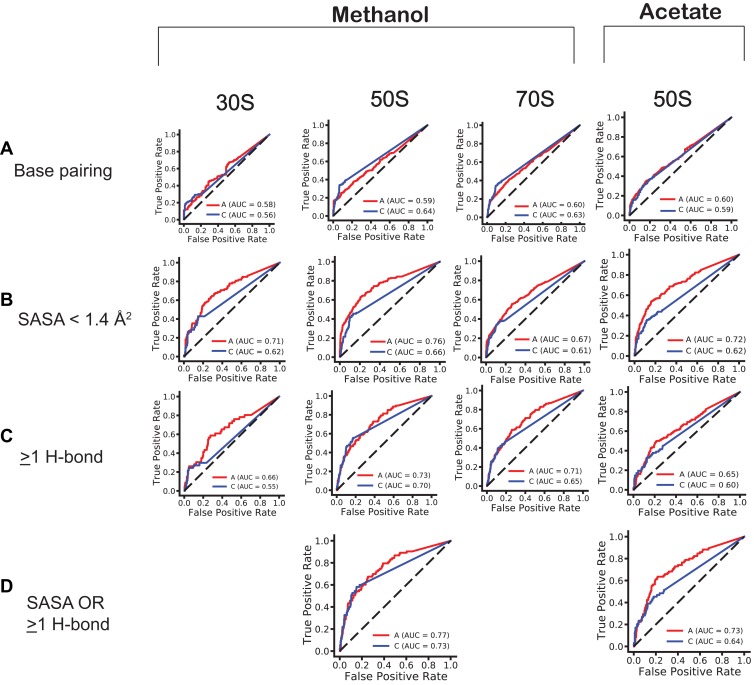
**Ribosome structural features and *in vivo* DMS reactivity.***A*-*C*, ROC curves that test how well base pairing, SASA, and ≥ 1 hydrogen bond near the WCF face impact DMS reactivity in the 30S, 50S, and 70S structures in methanol and acetate substrates. *D*, combinatorial ROC curves that combine SASA OR ≥ 1 hydrogen bond on DMS reactivity for the 50S in methanol and acetate substrates. SASA and distance cutoffs of < 1.4 Å^2^ and ≤ 3.5 Å, respectively, were applied. DMS, dimethyl sulfate; SASA, solvent accessible surface area; ROC, receiver operating characteristic.

The above sections revealed trends of DMS reactivity on SASA and on the number of hydrogen bonds for the two growth substrates. To judge the agreement of our ribosome structures with their *in vivo* folds, we assessed the area under the curve (AUC) from receiver operating characteristic (ROC) curves using the dependence of *in vivo* DMS reactivity thresholds on various binaries comprised of parameters extracted from our ribosome structures: base pairing, SASA, and number of hydrogen bonds (Table S5). In ROC plots, an AUC of 1.0 represents total agreement between the structural features and *in vivo* DMS reactivity, while a value of 0.5 indicates that the model with the structural feature and DMS reactivity is no better than random chance. Figure 5 provides a series of plots of true positive rate *versus* false positive rate for the DMS-reactive nucleotides A and C for 30S, 50S, and 70S grown with methanol and for the 50S with acetate.

We begin by considering separate ROCs for base pairing, SASA, and number of hydrogen bonds. The first ROC curves look at *in vivo* DMS reactivity as a function of rRNA base pairing, which was extracted computationally from the cryo-EM structures (see Experimental procedures). These curves showed only modest AUCs for 30S, 50S, and 70S ribosomes for methanol-grown and 50S for acetate-grown, with values ranging from 0.58 to 0.60 for A and from 0.56 to 0.64 for C with especially poor AUCs for the 30S ribosome (Fig. 5*A*). These findings suggest that base pairing alone does not capture DMS reactivity. Several bases had very low to no reactivity (Figs. S7 and S8) despite absence of base pairing for many (about 70%) of them. To assess the influence of such points, we filtered out reactivities <0.2 from the base pairing ROC curves. As shown in Fig. S9*A*, such filtering markedly improved the AUCs, with values ranging from 0.60 to 0.67 for A and from 0.64 to 0.82 for C in either growth condition. This improvement in AUC with filtering suggested that many of the low reactivity bases were in fact unpaired, *i.e.* false negatives (FNs).

Next, we considered ROC curves that evaluated *in vivo* DMS reactivity as a function of SASA, which was motivated in part by the FNs suggested from considering base pairing alone. It is important to note that we did not filter out any of the reactivities when plotting the SASA ROC curves. The SASA ROC curves showed markedly improved AUCs over the base pairing ROC curves for each subunit and for the entire ribosome, with values ranging from 0.67 to 0.76 for A and from 0.61 to 0.66 for C across all units and both growth substrates (Fig. 5*B*), suggesting that SASA alone explains *in vivo* DMS reactivity much better than base pairing alone, especially for A. The greater improvement for A is consistent with 558 out of 759 A’s being unpaired (74%) *versus* just 210 out 716 C’s being unpaired (29%) in the 50S subunit. The improved performance of SASA ROC curves over base pairing ROC curves also likely reflects the massive scale of the ribosome and its globular architecture. It also reflects the quality of our structures, with the best AUCs found for the highest resolution structure (*i.e.* for 50S in methanol-grown cells).

We then considered ROC curves that evaluate *in vivo* DMS reactivity as a function of the number of any hydrogen bonds near the WCF face, computationally extracted from the structures. This provides an orthogonal approach to SASA to account for FNs, as not all hydrogen bonding on the WCF face necessarily comes from canonical base pairing. Like with SASA ROCs, we did not filter out any reactivities in making the H-bond ROCs. These curves were calculated for ≥1 hydrogen bond near the WCF face (Fig. 5*C*) as well as for ≥2 and ≥ 3 hydrogen bonds at/near the WCF face (Fig. S9*B*). As described in the Experimental procedures, we included the N3 of A in the H-bond count as it improved the AUC for hydrogen bonding ROCs, suggesting that interaction on the sugar edge of the base can reduce DMS reactivity. The ROC curves with ≥1 hydrogen bond showed improvement in AUCs *versus* base pairing ROC curves for all cases except C in the 30S ribosome, with values ranging from 0.65 to 0.73 for A and from 0.60 to 0.70 for C across both growth substrates (Fig. 5*C*), which is similar to SASA alone. ROC curves that considered ≥2 and ≥ 3 hydrogen bonds at/near the WCF face performed fairly similarly to ≥1 hydrogen bond (Fig. S9*B*). These observations suggest that accounting for at least one hydrogen bond at/near the WCF face explains the *in vivo* DMS reactivity better than base pairing alone and comparably to SASA alone.

The improvement in ROC curves for SASA alone and hydrogen bonding at/near the WCF face alone inspired construction of combinatorial ROC curves that combine these two metrics (Fig. 5*D*). In these calculations, the binary had Boolean constraints of either the SASA <1.4 Å^2^ OR number of hydrogen bonds ≥1, 2, or 3 to give absence of reactivity. ROC curves with combined SASA OR ≥1 hydrogen bonding constraints showed somewhat better AUCs than the ones determined for SASA-alone or ≥1 hydrogen bond-alone, with values for 50S of 0.77 and 0.73 for A and C in the methanol condition, and 0.73 and 0.64 for A and C in the acetate condition. Other combinatorial ROC curves, either for SASA OR ≥2 or ≥3 hydrogen bonds for methanol or acetate, did not show improvement over SASA OR 1 hydrogen bond combinatorial ROC (Fig. S9*C*). We also note that using the Boolean of AND gave slightly lower AUCs (Fig. S9*D* and Table S5) than the SASA only condition.

In sum, the high quality of the ROC AUCs that combine SASA and hydrogen bonding support the *in vivo* relevance of the ribosome structures solved herein. In addition, the similarity of the combinatorial ROC AUCs for 50S between growth substrates further supports the overall similarity of the ribosome structure in these two conditions.

### Dimerization of the 50S subunit of *M. acetivorans* ribosome by the MRDF

**Figure 6:**
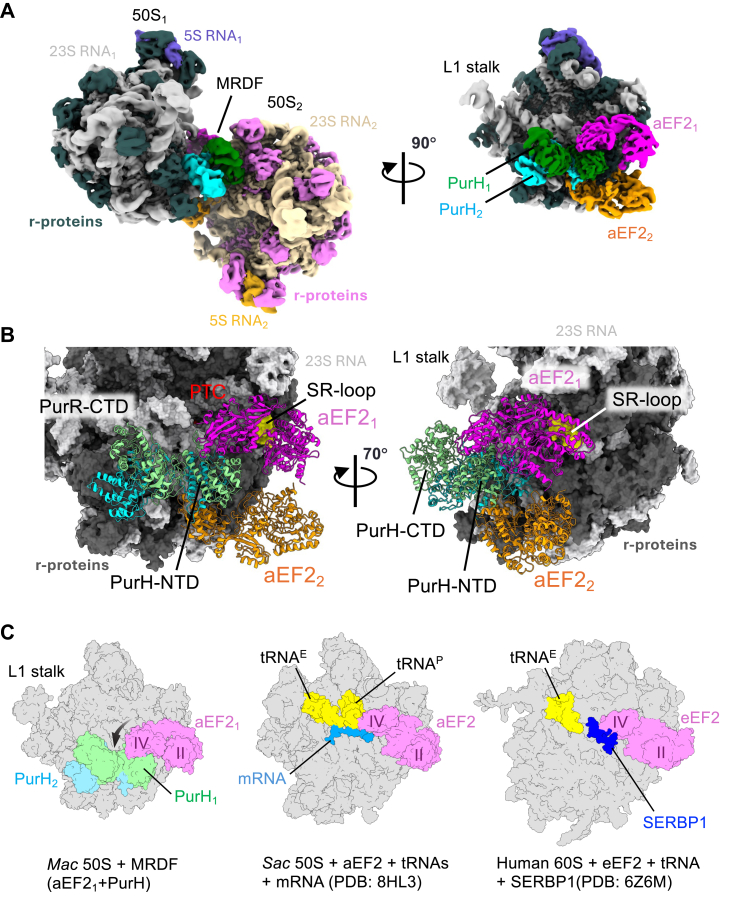
**Dimerization of the***Methanosarcina****acetivorans* ribosome by MRDF**. *A*, cryo-EM map of the *M. acetivorans* ribosome 50S subunit dimer formed by the methanogen ribosome dimerization factor (MRDF), showing the organization of rRNAs and r-proteins (50S subunits), and the MRDF components PurH and aEF2. In the *right panel*, one 50S subunit is removed to reveal MRDF binding on the surface of the adjacent 50S subunit. The peptidyl transferase center (PTC) and L1 stalk are indicated. *B*, close-up view of MRDF bound to the 50S subunit. Domain organizations of PurH and aEF2, as well as the locations of the PTC and sarcin–ricin loop (SR loop) of the 23S rRNA, are highlighted. *C*, structural comparison of aEF2/eEF2 associated with ribosomal large subunits from archaea and eukaryotes. *Left*: aEF2 bound to the 50S subunit as part of MRDF (this study). Movement of the domain IV of aEF2 for the MRDF formation relative to aEF2 as translation factor is indicated by a *black arrow*. *Middle*: aEF2 engaged with the 50S subunit during translation to mediate tRNA translocation. Bound tRNAs at the E and P sites and associated mRNA are indicated; the 30S subunit is omitted for clarity. *Right*: eEF2 associated with the 60S subunit of the human ribosome in complex with SERBP1 and E-site tRNA, forming a hibernating state. The 40S subunit is removed for clarity. All three ribosomes are oriented with the PTC at the center and the 5S rRNA at the *top*.

Cryo-EM data processing of the *M. acetivorans* ribosomes from methanol-grown cells revealed a minor subset of ribosome particles (∼10%) that form a 50S dimer (Figs. 6*A* and S10, *A* and *C*). This type of ribosome dimerization is unique compared with previously reported dimerization of ribosomes that primarily contain 70S ribosomes in bacteria and 80S ribosomes in eukaryote (51). Further analysis revealed that the dimer is stabilized by large, previously uncharacterized macromolecules situated between the 50S subunits. We designate this complex as the MRDF, which displays twofold symmetry and an elongated X-shape, measuring ∼155 Å in length and ∼74 Å in width.

To overcome anisotropic cryo-EM density caused by the elongated MRDF, we collected cryo-EM data at both 0° and 25° tilt angles and applied C2 symmetry during the single particle reconstruction (Fig. S10, *B* and *D*). This approach yielded an isotropic 3.5 Å resolution map, enabling confident model building. Using ModelAngelo (52), we identified MRDF as a heterotetramer composed of two copies of the purine biosynthesis enzyme PurH (bifunctional phosphoribosylaminoimidazolecarboxamide formyltransferase/5′-inosine monophosphate cyclohydrolase, MA4012) and two molecules of archaeal translation elongation factor 2 (aEF2, MA1257) (Fig. 6, *A* and *B*, Movies S3 and S4). The shorter axis of MRDF consists of the PurH dimer, while the longer axis is formed by two aEF2 molecules. The N-terminal domains of PurH (PurH-NTDs) mediate dimerization, and each PurH monomer associates with one aEF2 molecule, forming a heterotetramer. Notably, the two aEF2 molecules do not interact directly.

PurH is a bifunctional purine biosynthesis protein, playing a role in the *de novo* purine biosynthesis pathway by catalyzing the final two steps leading to the formation of inosine monophosphate from 5-aminoimidazole-4-carboxamide ribonucleotide. Structurally, PurH is organized into two distinct functional domains: the N-terminal cyclohydrolase domain (PurH-NTD), which carries out the inosine monophosphate cyclohydrolase reaction, and the C-terminal transformylase domain (PurH-CTD), which mediates the transfer of the formyl group from N10-formyltetrahydrofolate to the substrate 5-aminoimidazole-4-carboxamide ribonucleotide. Both domains are responsible for mediating PurH dimerization, which is essential for its enzymatic functions (53). Notably, in the structure determined in this work, the active sites of both domains are empty.

aEF2 is a G-protein that plays a crucial role in the translation cycle by facilitating the translocation of tRNAs and mRNA through the ribosome following peptide bond formation. This process is tightly regulated by the binding and hydrolysis of GTP, which induces conformational changes necessary for ribosome movement (54). aEF2 binds to the sarcin–ricin loop (SR-loop) of the 50S ribosome (Fig. 6*B*), a critical region for GTPase activation (55). The MRDF is anchored to both 50S subunits through multiple interfaces: the PurH dimer interacts *via* both its NTD and CTD with the 50S surface, while each aEF2 binds the sarcin–ricin loop of the adjacent 50S subunit. This architecture occludes 30S subunit binding, rendering the 50S subunit inactive for translation and suggesting a role in ribosome hibernation (Fig. 6*C*).

## Discussion

*M*. *acetivorans* is a valuable model organism for investigating methanogenesis, reverse methanogenesis, and archaeal metabolic diversity, particularly due to its ability to utilize a wide range of energy sources (20). Its mesophilic, nonhalotolerant nature makes it especially suitable for studying conserved and divergent features of archaeal translation as well as relevant to both archaeal and eukaryotic systems. In this study, we present the cryo-EM structure of the *M*. *acetivorans* ribosome, the first reported structure from a methanogenic mesophilic archaeon, which provides insights into the ribosome's architecture under physiologically and ecologically relevant conditions and its evolutionary divergence from thermophilic archaea.

### Architecture of a mesophilic archaeal ribosome

Our findings reveal that the *M*. *acetivorans* ribosome retains a complete set of ribosomal proteins conserved across the domain Archaea, along with their eukaryotic homologs, making it a suitable model for investigating translation mechanisms common to the domains *Archaea* and *Eukarya* (Fig. 3*A* and Table S2). In contrast, thermophilic archaeal ribosomes often carry additional r-proteins likely required for rRNA stabilization at elevated temperatures. These proteins, though peripheral to the ribosome core, may enhance structural integrity under thermal stress. Furthermore, these additional r-proteins are retained in the Asgard kingdom of the domain *Archaea*—the closest known relatives of eukaryotes—and in eukaryotic ribosomes themselves (13). It is proposed that such thermophilic adaptations of ribosomes using extra r-proteins and these extra proteins may have been used during eukaryotic ribosome evolution for translation regulation or ribosome localization (56, 57).

The correlation between rRNA G + C content and optimal growth temperature further supports a role for nucleotide composition in stabilizing rRNA secondary and tertiary structures (Fig. 3*D* and Table S3). Mesophilic archaeal species, including *M*. *acetivorans*, consistently exhibit lower rRNA G + C content; in contrast, the G + C content of genomic DNA does not show a clear correlation with their growth temperatures. This difference may be explained by the fact that DNA maintains its double-stranded shape in thermophiles using various stabilizing mechanisms such as binding archaeal histone or architectural protein and introducing positive supercoiling (58). Because of the extensive unpaired regions, rRNA, on the other hand, relies primarily on its sequence and secondary/tertiary structure as well as r-protein binding to maintain stability, particularly under high-temperature conditions, making elevated G + C content a more critical factor for its structural integrity. Mesophilic archaeal species, including *M*. *acetivorans*, consistently exhibit lower rRNA G + C content, which may contribute to greater RNA flexibility and may facilitate protein translation at moderate temperature in contrast to thermophiles (*e.g.*, *S*. *acidocaldarius* ∼75 °C, *T*. *kodakarensis* ∼85 °C, and *P*. *furiosus* ∼95 °C). A correlation between G + C content and optimal growth conditions was reported by our group previously and required hierarchical and cooperative folding of RNA, where tertiary structure interactions equal or exceed those of secondary structure interactions (38), a phenomenon that in the case of ribosomes can be assisted by r-proteins. Thus, the *M*. *acetivorans* ribosome is well suited for studying translation with biochemical and biophysical assays, including single-molecule fluorescence experiments.

### Connecting orthogonal methods for structural elucidation in *M. acetivorans*

Chemical probing of RNA structure has become an increasingly important approach in judging the *in vivo* relevance of structures and capturing RNA dynamics (42, 59). Our comparative cryo-EM analysis of ribosomes isolated from methanol- and acetate-grown cells revealed nearly identical structures of rRNAs, consistent with our previous *in vivo* DMS probing study (27). This observation was supported by the lack of dependence of differences in DMS reactivity on differences in SASA or number of hydrogen bonds, and by ROC curves that were very similar in both growth substrates (Figs. 5*D*, S7*C*, and S8*C*), although subtle chemical accessibility differences can be detected at specific sites in rRNA, such as those observed in the greater changes in SASA (<−10 and > 10 Å^2^) and changes in number of hydrogen bonds (<−1 and > 1 bond) (Figs. S7*C* and S8*C*). The variation between the differential DMS reactivity results and the 3D structure may arise because single-particle cryo-EM relies on averaging many ribosome particles (60,000–200,000) to reconstruct a high-resolution 3D map and structure, thereby capturing the most dominant states of ribosome. In contrast, DMS probing reports nucleotide-level reactivity in individual rRNA molecules across the population, allowing detection of rare or transient heterogeneity, which may be due to ribosome dynamics or assembly (46, 47, 49, 60, 61). Variation between differential DMS reactivity and structure is reflected in AUCs for ROC curves that have maximal values of only ∼0.75, which may reflect the inability to capture false positives (*i.e.* bases with SASA of 0 Å^2^ that still react with DMS *in vivo* owing to fluctuations or assembly (Fig. 4*B*), as reflected by points along the y-axis in Fig. S7, *A* and *B*). Despite this, the static 3D structures offer important information on the tertiary nature of the rRNA bases and how they influence DMS reactivity, making cryo-EM and DMS probing studies complementary to providing insights into ribosome structure and dynamics.

### Ribosome dimerization and hibernation in *M. acetivorans*

This study identifies the first archaeal ribosome dimerization factor that uniquely targets the 50S subunit (Fig. 6) rather than the intact 70S ribosome (51). MRDF represents a novel paradigm in ribosome regulation, as it is composed of multifunctional proteins—PurH and aEF2—that are independently essential for purine biosynthesis and translation elongation, respectively. This repurposing of core metabolic and translational components distinguishes MRDF from previously described dedicated ribosome dimerization factors (51, 62).

The structural inactivity of both PurH and aEF2 in the dimerized state—supported by empty active sites—suggests that MRDF formation may occur under nutrient-limited conditions, where both purine biosynthesis substrate and GTP levels are low. This implies a dual-sensing role: PurH as a nucleotide metabolism sensor and aEF2 as a GTP sensor, coordinating cellular metabolism with new protein synthesis. Such a mechanism would be metabolically efficient, linking global translation suppression to nutrient availability. The MRDF-induced dimerization physically blocks 30S subunit association, locking the 50S ribosomes in an inactive state (Fig. 6). This structure likely represents a hibernation intermediate, allowing *M. acetivorans* to regulate translation without the need for dedicated inhibitory proteins.

Our findings also raise the possibility that 50S dimerization, rather than 70S pairing, may be a conserved or underappreciated mode of ribosome hibernation in archaea. Strikingly, aEF2—a core component of MRDF—has a eukaryotic counterpart, eEF2, which was also identified as a ribosome hibernation factor in vertebrate systems such as zebrafish and *Xenopus* eggs (30). In these systems, eEF2 participates in stabilizing translationally inactive ribosomes in the egg and also early embryo, emphasizing that elongation factor-based ribosome hibernation may be a deeply conserved mechanism. In human cells, eEF2 has also been observed to inactivate 80S ribosomes together with SERBP1, a dedicated hibernation factor, forming translationally repressed complexes under stress conditions (Fig. 6*C*) (63). These findings support a model in which eukaryotic ribosome hibernation relies on eEF2 in combination with dedicated hibernation-specific proteins, whereas the archaeal system described here relies solely on the refunctionalization of an essential metabolic (PurH) and translational factor (aEF2) without auxiliary inhibitors (Fig. 6).

Our structural observation of aEF2-mediated ribosome inactivation in archaea suggests that this mode of regulation predates the divergence of eukaryotes and may represent an ancestral mechanism of ribosome hibernation (Fig. 6*C*). In contrast, the bacterial homolog of EF2, known as EF-G, has not been implicated in ribosome hibernation (62). This distinction indicates that aEF2/eEF2-based hibernation likely emerged after the evolutionary separation of *Archaea* and *Bacteria* from the last universal common ancestor, underscoring the importance of studying archaeal ribosomes to elucidate new insights into protein translation in the domains *Archaea* and *Eukarya*.

Future investigations will explore the biological function of MRDF-mediated *M. acetivorans* ribosome dimerization across different growth phases (*e.g.*, logarithmic versus stationary) and metabolic conditions (*e.g.*, methanol *versus* acetate as energy sources), as well as its regulation under environmental stresses. These studies will enhance our understanding of translational regulation in methanogens and expand the known repertoire of ribosome hibernation mechanisms in all three domains of life.

## Experimental procedures

### Growth conditions for *M. acetivorans* cells

*M. acetivorans* was cultured as described (27, 64) with slight modifications. Cells were grown in a high-salt medium buffered with 45 mM HEPES (pH 6.8) and supplemented with either 100 mM sodium acetate or 100 mM methanol as the growth substrate. The high-salt medium contains: 23.4 g/L NaCl, 1.0 g/L KCl, 11.0 g/L MgCl_2_, 0.2 g/L CaCl_2_, 1% (v/v) vitamin solution, 1% (v/v) trace mineral solution (65), and 0.001% (w/v) resazurin as a redox indicator. The medium was continuously sparged with a 95% N_2_/5% H_2_ gas mixture while stirring overnight in an anaerobic chamber. After overnight stirring, NH_4_Cl (1.0 g/L) and cysteine-HCl monohydrate (0.5 g/L) were added as the nitrogen source and reducing agent, respectively, and stirred under anaerobic conditions. A total of 800 ml of medium was aliquoted into 1 L glass bottles sealed with black rubber stoppers fitted with glass tubing to equalize pressure during autoclaving. Bottles were covered in aluminum foil and autoclaved at 121 °C for 30 min. After autoclaving, the media were cooled to room temperature and returned to the anaerobic chamber overnight under the same 95% N_2_/5% H_2_ atmosphere to ensure complete anaerobiosis. For small-scale cultures, 100 ml aliquots were dispensed into 100 ml glass bottles, sealed with blue butyl rubber stoppers and aluminum crimps prior to autoclaving. These bottles were prepared on the same day as media bottling to ensure consistency in growth conditions.

For anaerobic cultivation, sterile stocks of 2.5% (w/v) Na_2_S·9H_2_O (10 ml/L) and 1 M KH_2_PO_4_ (5.0 ml/L) were added to the prepared media under anaerobic conditions. Cultures were supplemented with either 100 mM methanol or 100 mM sodium acetate. Media were inoculated with *M. acetivorans* cells at 1% (v/v) for growth with methanol and 10% (v/v) for growth with acetate. Methanol-grown cells were cultured in 800 ml volumes, while acetate-grown cells were cultured in 100 ml volumes. Cells were grown to mid-exponential phase as described (32).

### Ribosome purification

Methanol- and acetate-grown *M. acetivorans* cells were harvested and washed in resuspension buffer containing 20 mM Tris-OAc (pH 7.5), 60 mM NH_4_Cl, 7.5 mM MgCl_2_, and freshly added 6 mM β-mercaptoethanol. Cells harvested from either condition were resuspended in 800 μl of the same buffer and lysed by sonication on ice using a Branson Fisher Scientific 150E Sonic Dismembrator (eight cycles at 15% amplitude, 10 s on, 1 min off). For acetate-grown cells, due to lower biomass, cells were sonicated using three cycles under identical sonication cycles. The crude lysate from either condition was clarified in 1.7 ml microcentrifuge tubes by centrifugation at 16,000*g* for 20 min at 4 °C. The supernatant was transferred to a fresh tube, and the remaining pellet was subjected to a second round of centrifugation under the same condition to pool supernatants. For ribosome pelleting, 100 μl of the clarified lysate was layered onto 400 μl of sucrose cushion buffer (20 mM Tris-OAc, pH 7.5; 500 mM NH_4_Cl; 10 mM MgCl_2_; 37.7% w/v sucrose; and 6 mM fresh β-mercaptoethanol) in Beckman polycarbonate centrifuge tubes. Samples were centrifuged at 85,000*g* for 2 h at 4 °C. Pellets were gently washed and resuspended in buffer, then pooled and concentrated using Amicon Ultra centrifugal filters with a 3 kDa molecular weight cutoff. Ribosome concentration was determined using A260 and A280 readings, the rRNA quality was assessed by agarose gel electrophoresis, the presence of r-proteins was determined by mass spectrometry, and the initial screening of ribosome particles was done by negative staining.

### Cryo-EM grid preparation and data acquisition

Quantifoil grids (R1.2/1.3 Au 300 mesh) were glow-discharged for 30 s using a PELCO easiGLow with vacuum level set at 0.39 mBar, and discharge current at 15 mA and negative polarity. Subsequently, 3.5 μl of a 0.5 mg/ml sample was applied to the grids, followed by immediately plunge-freeze in liquid ethane using an FEI Mark IV Vitrobot set to 100% chamber humidity and maintained at 10 °C. The frozen grids were screened by an FEI Talos Arctica G2 microscope operating at 200 kV at Penn State Huck Institutes of the Life Science Cryo-EM Facility. Imaging was performed using the Falcon 4 direct electron detector with aberration-free image shift enabled. Data collection was conducted for ribosome prepared from methanol-grown cells, without and with 25ᵒ degree tilted stages at a nominal magnification of x,190,000 and a pixel size of 0.733 Å, a total electron dose of 50 e^−^/Å^2^, and with defocus values ranging from −0.6 μm to −2.0 μm. Automatic data acquisition was carried out using EPU (FEI, Thermo Fisher Scientific), resulting in 6631 movies without tilting and 7750 movies with tilting. The cryo-EM data (4119 movies) of ribosome prepared from acetate-grown cells were collected using FEI Talos Krios G3 operating at 300 kV with a Falcon 4 detector at Penn State Huck Institutes of the Life Science Cryo-EM Facility. The nominal magnification was set at x59,000 and a pixel size of 1.1 Å, a total electron dose of 50 e^−^/Å^2^, and defocus values ranging from −0.8 μm to −2.0 μm.

### Cryo-EM data processing

All data were processed using CryoSPARC v4.1.2 (66) and the detailed workflows are illustrated in Supplemental Data. The movies were aligned and dose-weighted using patch motion correction, and patch-contrast transfer function estimation across the micrographs. Automatic particle picking was performed on 100 micrographs with a particle diameter range from 200 Å to 300 Å. After particle extraction, two rounds of 2D classification showed well-defined features of the 70S and 50S ribosomes. Selected 2D classes were used as templates for particle picking from all micrographs, with an assigned particle diameter of 220 Å. The extracted particles were used for multiple rounds of 2D classifications followed by generating initial volumes of the 50S ribosome and 70S ribosome by reference-free *ab initio* 3D map reconstruction. These *ab initio* maps were used to perform a series of 3D refinements, yielding the final cryo-EM maps of the 50S subunit of ribosome and 70S ribosome. Final 3D reconstructions were obtained at a nominal resolution of 2.92 Å for the 70S ribosome and 2.38 Å for the 50S subunit from methanol-grown cells. In addition, the 50S subunit from acetate-grown cells was resolved to 2.85 Å. For 50S ribosome dimer with MRDF, Fourier cropped particles (from 800 to 600 pixel box size, image pixel size = 0.977 Å) are used for all classification steps.

### Model building and refinement

Initial models of the *M. acetivorans* ribosome were built by AlphaFold3 (67) and fitted into cryo-EM maps using Namdinator (68), Isolde (69), and Coot v0.9.8.95 (70) followed by refining the structures by Phenix v1.20.1 (71). Positions of Mg^2+^ and K^+^ atoms in the *M. acetivorans* ribosome were found using the atomic resolution cryo-EM structures of the *E. coli* ribosome (PDB: 9D89, 7K00) (72, 73) and the *Pyrobaculum calidifontis* ribosome (PDB: 9E71) (74) as references. Nucleoside modifications were manually built into structures using Coot. Structures were refined by Phenix with secondary structure restraints, and validated by MolProbity (75) (Table S1).

Using 3.5 Å resolution cryo-EM map of the ribosome 50S subunit dimer with MRDF (Fig. 4), extract the cryo-EM map corresponding to MRDF by using ChimeraX and Coot, and used it for protein model building by ModelAngelo (52) without provided sequences followed by sequence search with all ORFs in the *M. acetivorans* genome. PurH (MA4012) and aEF2 (MA1257) showed E-scores of 7.59 x 10^-16^ and 5.75 × 10^-7^, respectively and the next top candidate showed an E-score of 0.01. Initial models of the *M. acetivorans* PurH and aEF2 were built by AlphaFold3 (67) and these models were manually fitted into the cryo-EM map together with the ribosome 50S subunit. The structure was refined by Phenix (71) with secondary structure and reference structure restraints from the methanol-grown 50S subunit (PDB ID: 9NTA) and non-crystallographic symmetry constraints (Table S1). EM map display and figure preparation were performed using USCF ChimeraX (76).

### Chemical probing data analysis, availability, and reproducibility

Structural analysis in conjunction with the DMS reactivity was performed using *M. acetivorans* 50S structures from methanol-grown (PDB ID: 9NTA) and acetate-grown (PDB ID: 9NRI) cells as well as the 70S structure from methanol-grown cells (PDB ID: 9O17). The published DMS reactivity files used in the analysis are available in GEO GSE229536 as “GSE229536_M_minus_M_plus_ln_nrm.react.txt.gz” for methanol and “GSE229536_A_minus_A_plus_ln_nrm.react.txt.gz” for acetate (27). Differentially reactive residues were calculated by first averaging the reactivity data from the three different gene IDs for 16S (929, 1667, and 4835), 23S (930, 1669, and 4834), and 5S (931, 1670, and 4833) at each A and C position. Then, methanol reactivity values were subtracted from acetate ones at each position to yield the differentially reactive values. As described previously, nucleotides that had values |Reactivity_Methanol_ - Reactivity_Acetate_| ≥ 0.5 were considered highly differentially reactive (27). Nucleotide numbering from that study is provided in Table S4.

### Calculating SASA of DMS reactive atoms

Solvent accessibility was calculated using the sasa_measure.py python script on the GitHub page. The tool was adapted from the Shrake-Rupley rolling-ball method to calculate solvent accessibility per atom of a nucleotide (77). Briefly, the parameters that were inputted in the script were the structure used (9O17 for methanol 16S rRNA, 9NTA for methanol 23S and 5S rRNA, and 9NRI for acetate 23S and 5S rRNA), the chain, which was AA (16S) from 9O17 and BA (23S), BB (5S) from 9NTA and 9NRI, a ball-size of 1.4 Å (77), and the number of points surrounding the atom was 400. The output file was parsed only for A(N1) and C(N3), since they are the DMS-reactive nitrogens (39). The DMS reactivity of the base was plotted against solvent accessibility of these atoms for each condition and shown in Fig. S7, *A*–*C* for all A and C in 23S and 5S and is noted in Table S4 for the differentially reactive A and C in 23S rRNA. The SASA calculation performed was also used to generate the ROC curves (Figs. 5 and S9).

### Protein-RNA contacts for highly reactive and differentially reactive nucleotides

Once the highly differentially reactive nucleotides were identified (Table S4), they were further analyzed using PyMOL’s command line. First, we identified the protein chains that were ≤ 4.0 Å away from the differentially reactive nucleotide. Second, the number of RNA atom contacts to each of these protein chains were identified and averaged for the two growth conditions. The python script that outputs protein chains and the number of atom contacts is ProteinRNA_contacts.py. Any proteins that had at least 10 RNA atom contacts were considered as proteins of interest in our analysis.

### Hydrogen bonding interactions for DMS reactive residues

We used Dissecting the Spatial Structure of RNA (DSSR) (44) to identify hydrogen bonding interactions, which were defined as having a distance of 2.0 to 3.5 Å between heteroatoms, of all As and Cs. The hydrogen bonding interactions were identified using the All_Hbonds_Counter.py script to output the number of H-bonding interactions for all As and Cs. Any hydrogen bonding that was considered “questionable” by DSSR was filtered out of the dataset.

### ROC curves

ROC curves were generated for 50S (23S and 5S rRNA) for both methanol and acetate conditions and for 30S and for 70S (23S, 16S, and 5S rRNA) only for methanol. Only DMS data for A and C were considered. Data for canonical WCF base pairs (AU and GC) or for hydrogen-bonding interactions were collected from DSSR (44). Hydrogen bonds from heteroatoms N6, N1, and N3 for adenosine and from N4, N3, and O2 for cytosine were considered for the hydrogen bonding parameter of the ROC curve. We found that including N3 improved the AUC values for A. The script to parse for hydrogen bonds on these atoms is Near_WCF_Counter.py, and the script to generate ROC curves is ROC.py.

## Use of large language and visualization models

Command line tools and graphical user interfaces such as DSSR of RNA (DSSR v.2) (44) and PyMOL Molecular Graphics System (PyMOL v. 3.1.0) were used to gather tertiary structure and visualize structural data respectively. Python (v. 3.14.0a6) was employed to color structures, filter and count structural features, and generate data frames for plotting. GGplot2 from RStudio (v.4.3.3) and related dependencies such as tidyverse (v.2.0.0) were used to generate plots. Codes were made with assistance from Virtual Studio Code (v. 1.97.1) and ChatGPT-4o.

## Data availability

All data needed to evaluate the conclusions of this paper are present in the Main Text and/or the Supplementary Materials. The cryo-EM maps and the refined models were deposited in the Electron Microscopy Data Bank (EMDB, www.ebi.ac.uk/emdb/) and PDB (www.rcsb.org): *M.acetivorans* 70S ribosome (EMD-49998/PDB:9O17), *M.acetivorans* 50S ribosome subunit from methanol-grown cells (EMD-49757/PDB:9NTA), *M.acetivorans* 50S ribosome subunit from acetate-grown cells (EMD-49734/PDB:9NRI), *M.acetivorans* 50S-50S subunits dimer with MRDF from methanol-grown cell (EMD-70864/PDB:9OU7). Published DMS reactivity files available at GEO GSE229536 were also used for analysis. Analysis for ROC plots, protein-RNA contacts, hydrogen-bonding interactions, and solvent accessible surface area are hosted in the GitHub page with the following link: [https://github.com/The-Bevilacqua-Lab/MAC_Ribosome_Analysis.git]. Additional data used to make bar graphs and scatter plots in the paper are hosted in FigShare with the following link: [https://figshare.com/s/8615f1879b41fdc40fdc].

## Supporting information

This article contains supporting information.

## Declaration of Generative AI and AI-Assisted Technologies in the Writing Process

ChatGPT was also used for grammatical correction of the text and to aid with coding.

## Conflict of interest

The authors declare that they have no conflicts of interest with the contents of this article.
