# Supplementary material for: Cryo-EM study and in vivo chemical mapping of the Methanosarcina acetivorans ribosome and its dimerization via a repurposed enzyme and translation factor
Source: J Biol Chem. 2025 Sep 4;301(11):110686. doi: 10.1016/j.jbc.2025.110686 (PMC12605026; doi:10.1016/j.jbc.2025.110686)
Supplement: Supporting information [file mmc1.docx]

**Supporting Information**

**Cryo-EM Study and *In Vivo* Chemical Mapping of the *Methanosarcina acetivorans* Ribosome and its Dimerization via a Repurposed Enzyme and Translation Factor**

George N.R. Fordjour^1-5^, Anwesha Ghosh^3,6^, James G. Ferry^1^, Jean-Paul Armache^1,2,4^, Philip C. Bevilacqua^1-3,6^, and Katsuhiko S. Murakami^1-3^

^1^Department of Biochemistry and Molecular Biology, Penn State University, University Park, PA 16802

^2^Center for Structural Biology, Penn State University, University Park, PA 16802

^3^Center for RNA Molecular Biology, Penn State University, University Park, PA 16802

^4^Center for Eukaryotic Gene Regulation, Penn State University, University Park, PA 16802

^5^Molecular Machines Mechanism and Structure Predoctoral Training Program, Penn State University, University Park, PA 16802

^6^Department of Chemistry, Pennsylvania State University, University Park, Pennsylvania 16802, USA

^7^These authors contributed equally

*Corresponding authors. Email: [kum14@psu.edu](mailto:kum14@psu.edu) and [pcb5@psu.edu](mailto:pcb5@psu.edu)

Keywords: ribosome, cryo-electron microscopy, archaea, methanogen, RNA chemical structure probing, structural biology

**
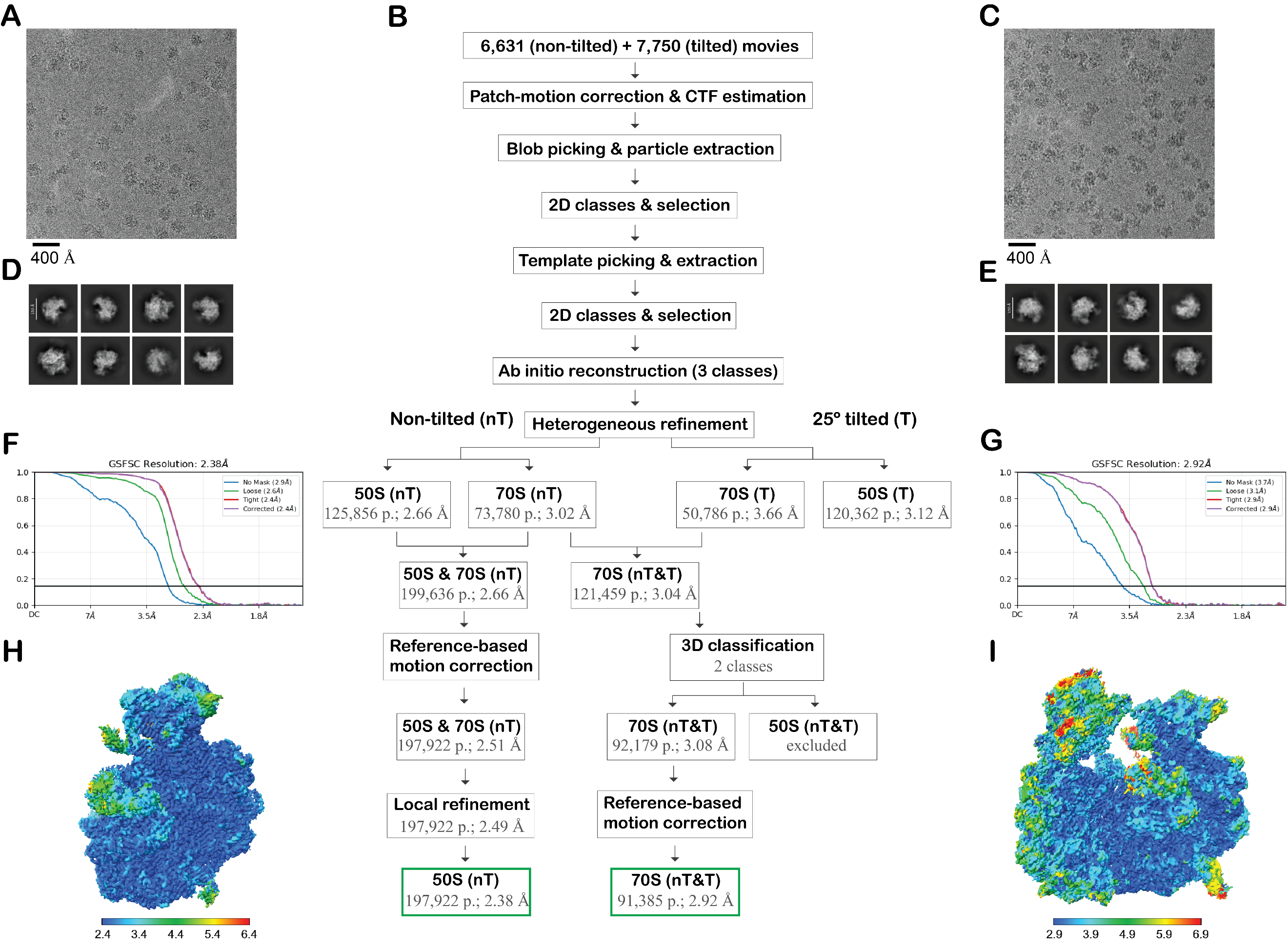
**

**Supplementary Fig. S1**: Cryo-EM data processing of the 50S subunit and 70S ribosome of methanol-grown *M. acetivorans* cells. (**A, C**) Representative micrographs showing particle distribution of (**A**) 50S subunit and (**C**) 70S ribosome. (**B)** Cryo-EM data processing and classification workflow. (**D, E**) Representative 2D class images of 50S subunit (**D**) and 70S ribosome (**E**). (**F, G**) Fourier shell correlation (FSC) plot for half-maps with 0.143 FSC criteria indicated nominal resolutions at 2.38 Å for 50S subunit (**F**) and at 2.85 Å for 70S ribosome (**G**). (**H, I**) Local resolution maps of the 50S subunit (**H**) and 70S ribosome (**I**).

**
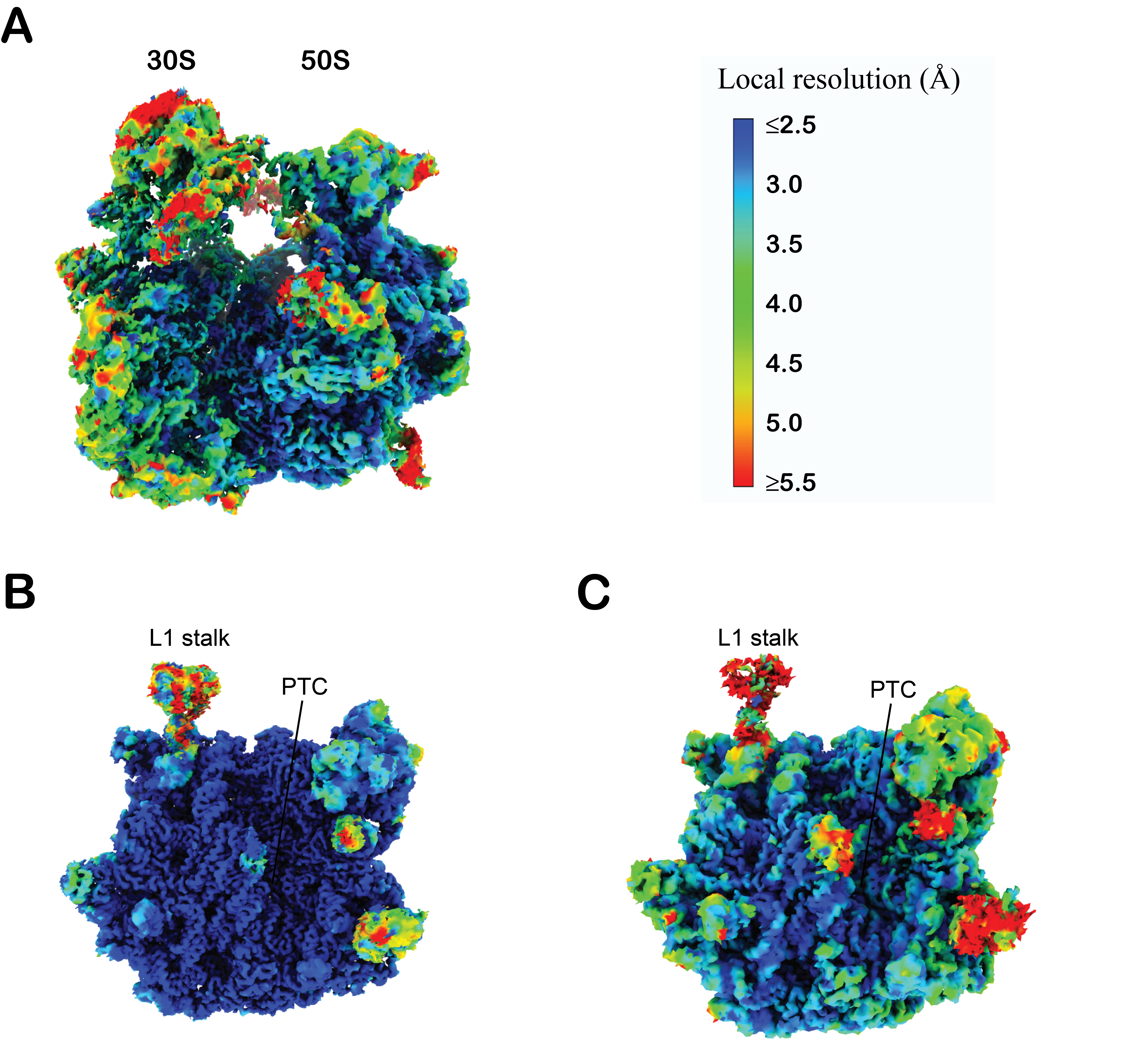
**

**Supplementary Fig. S2:** Local resolution maps of the *M. acetivorans* ribosome of (**A**) 70S ribosome from methanol-grown cells, (**B**) 50S subunit from methanol-grown cells, and (**C**) 50S subunit from acetate-grown cells. A color key for resolutions is provided.

**
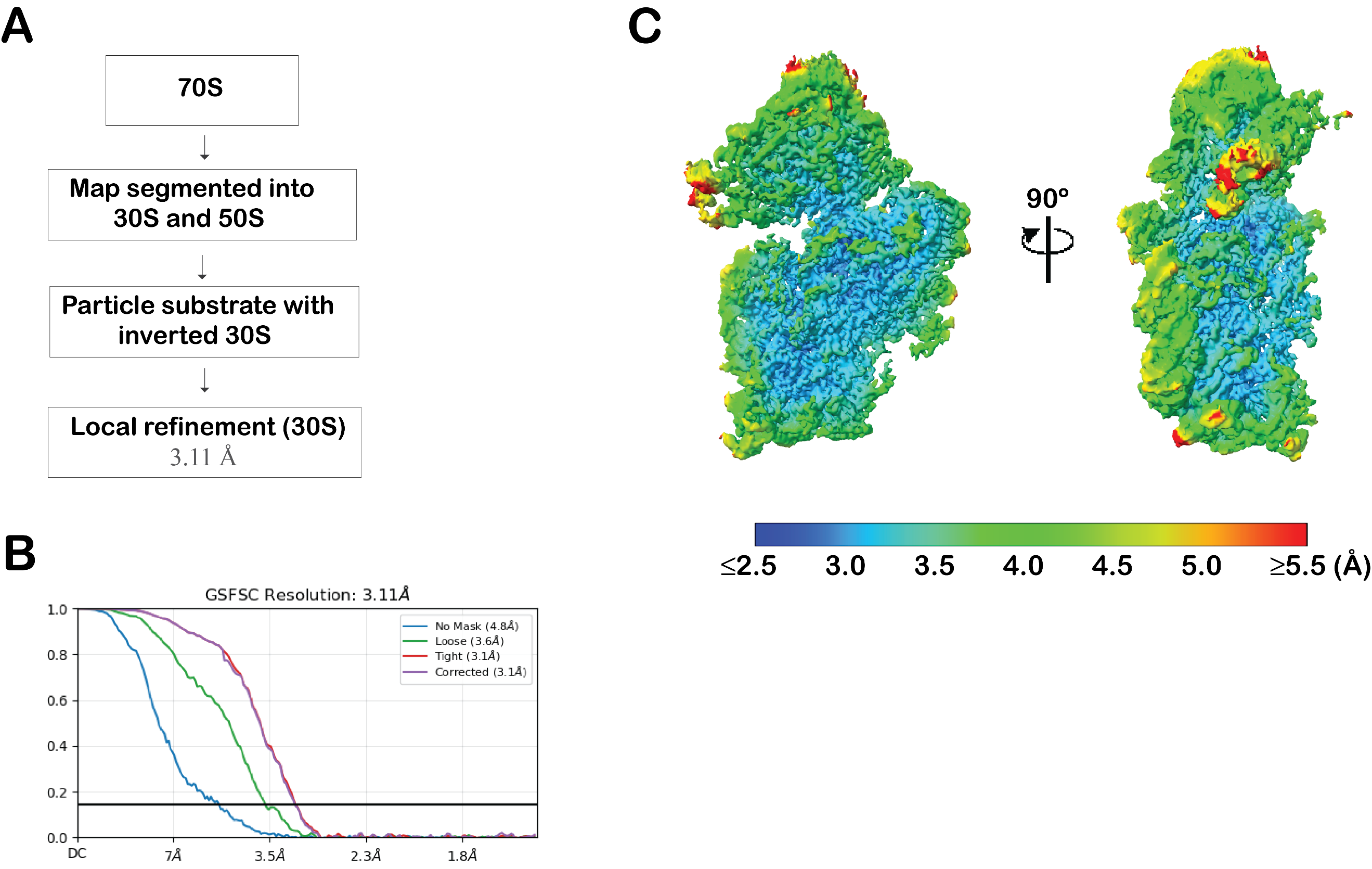
**

**Supplementary Fig. S3**: Cryo-EM data processing of the 30S subunit ribosome from methanol-grown cells. (**A**) Local refinement workflow. (**B**) Fourier shell correlation (FSC) plot for half-maps with 0.143 FSC criteria indicated nominal resolution at 3.11 Å. (**C**) Local resolution map.

**
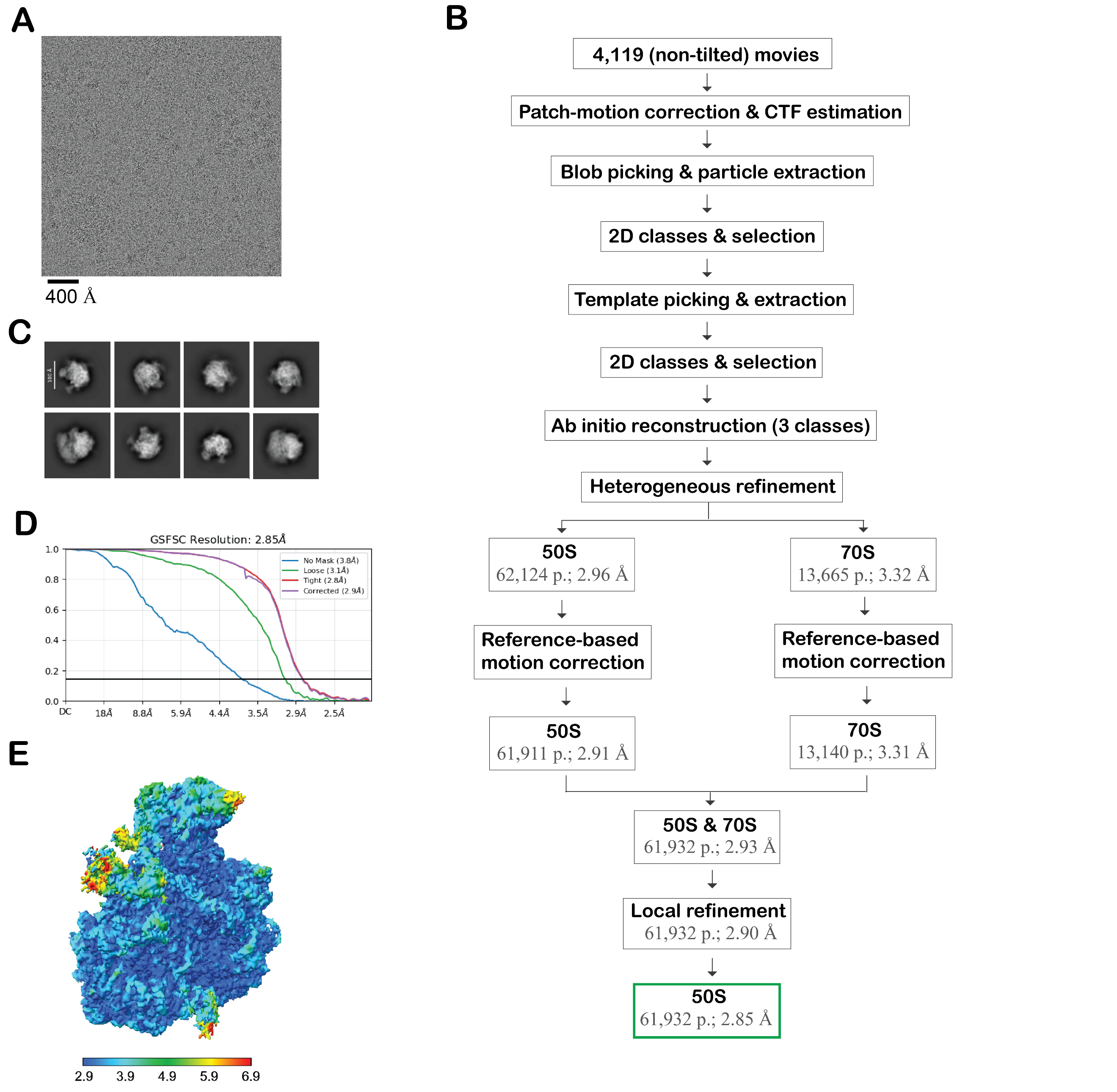
**

**Supplementary Fig. S4**: Cryo-EM data processing workflow for the 50S subunit ribosome from acetate-grown cells. (**A**) A representative micrograph showing particle distribution. (**B**) Cryo-EM data processing workflow. (**C**) Representative 2D class images. (**D**) Fourier shell correlation (FSC) plot for half-maps with 0.143 FSC criteria indicated nominal resolution at 2.85 Å. (**E**) Local resolution map.

**
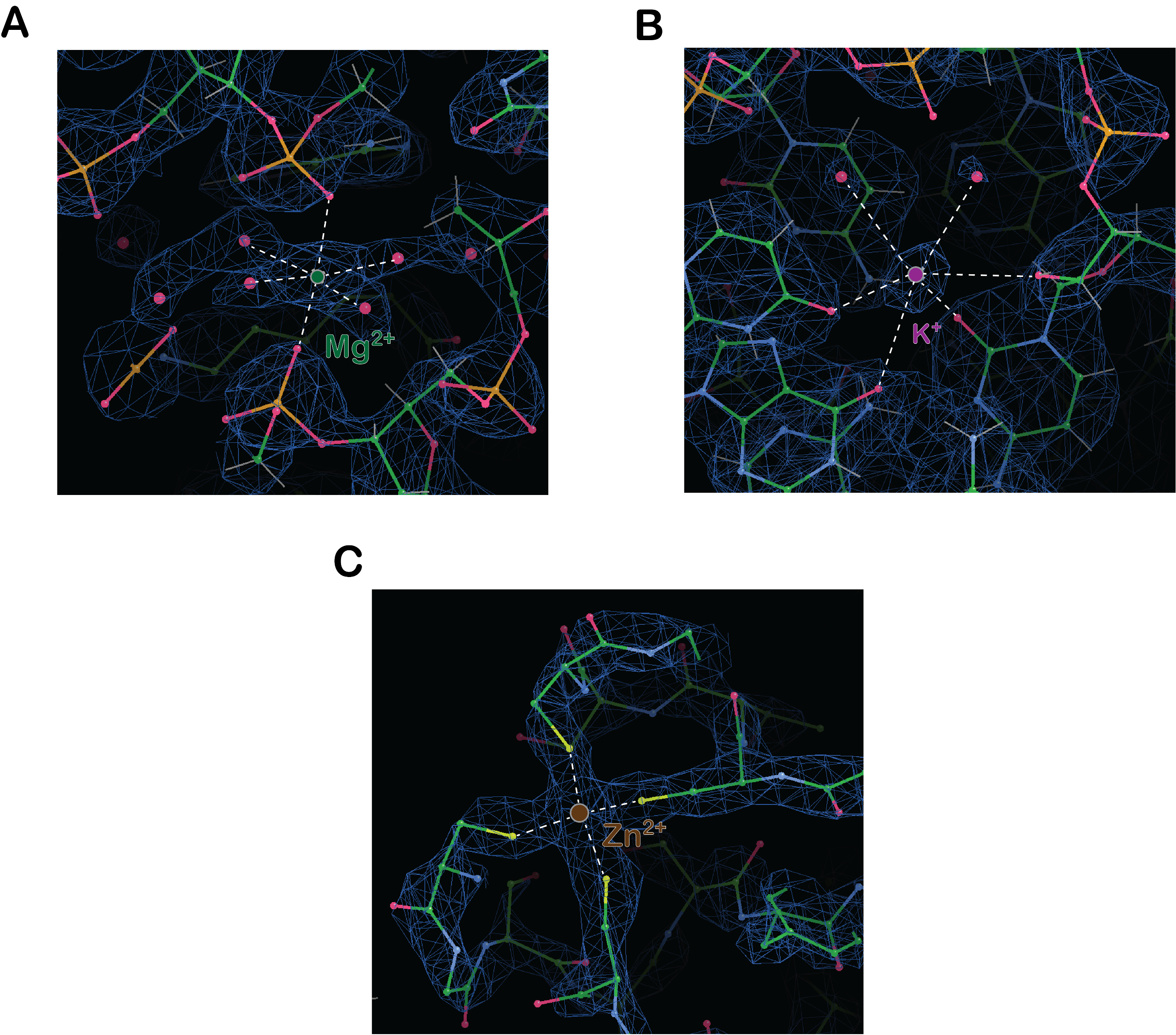
**

**Supplementary Fig. S5**: Representative coordination of metal ions (**A**, magnesium; **B**, potassium; **C**, zinc) in ribosome. rRNA (**A** and **B**) and r-protein (**C**) are depicted as stick models, with overlaid cryo-EM densities (blue mesh). Waters are depicted as red spheres. Coordination bonds are shown in dash lines.

**
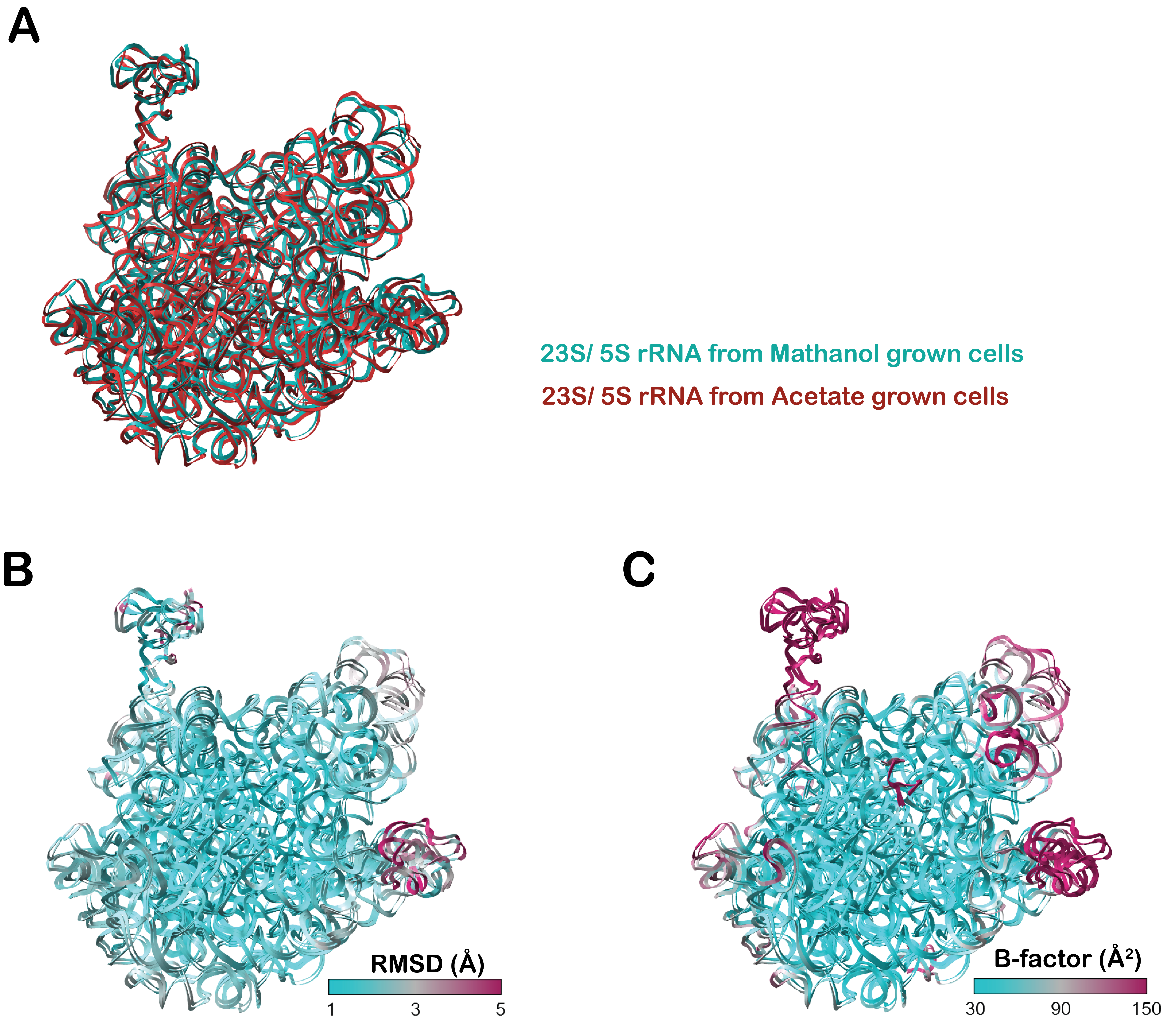
**

**Supplementary Fig. S6**: Structural comparison of the 50S rRNAs from methanol- and acetate-grown *M. acetivorans* cells. (**A**) Overlayed structures of 50S rRNAs from methanol- and acetate-grown *M. acetivorans* cells. (**B**) R.M.S.D. of 50S rRNAs from methanol- and acetate-grown cells. (**C**) B-factor distribution of the 50S rRNAs from methanol- and acetate-grown cells.

**
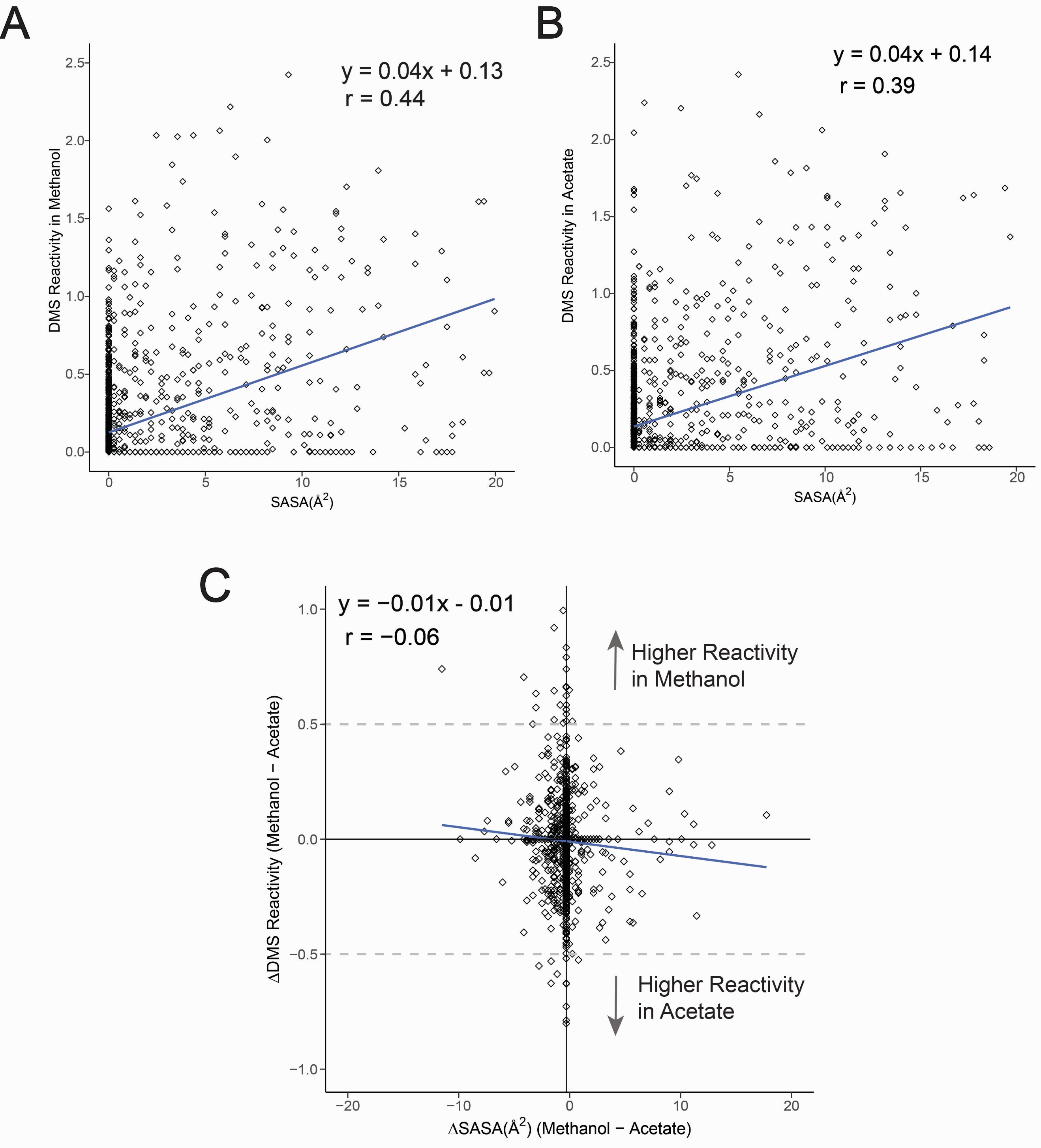
Supplemental Fig. S7.** Dependence of DMS reactivity on solvent accessibility surface area (SASA). (**A**, **B**) Scatter plots of DMS reactivity versus SASA for methanol and acetate, respectively. (**C**) Change in DMS reactivity (methanol minus acetate growth conditions) versus the change in SASA.

**
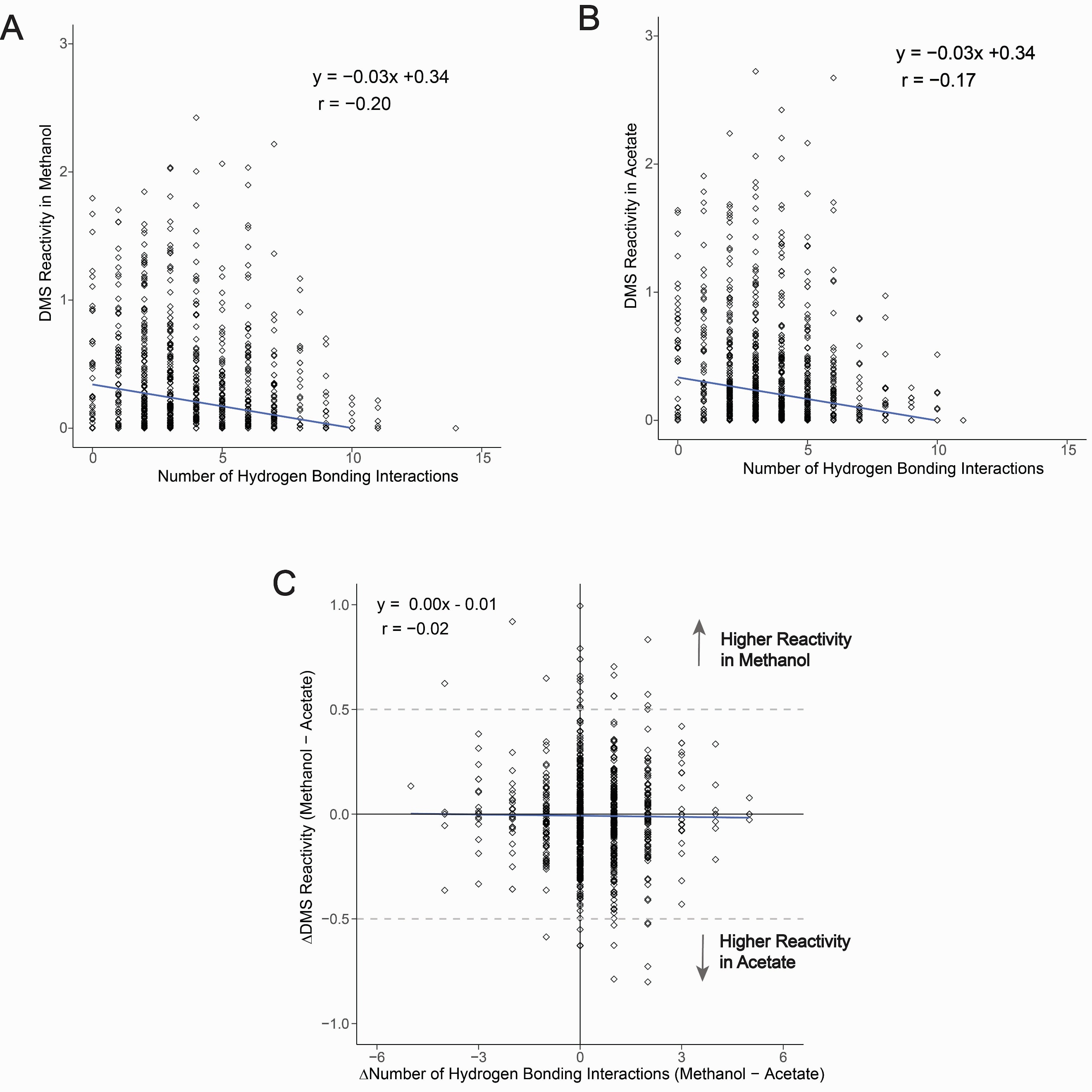
Supplemental Fig. S8:** Dependence of DMS reactivity on the number of hydrogen bonds. (**A**, **B**) Scatter plots of DMS reactivity versus number of hydrogen bonding interactions for methanol and acetate, respectively. (**C**) Change in DMS reactivity (methanol minus acetate growth conditions) versus change in number of hydrogen bonding interactions.

**
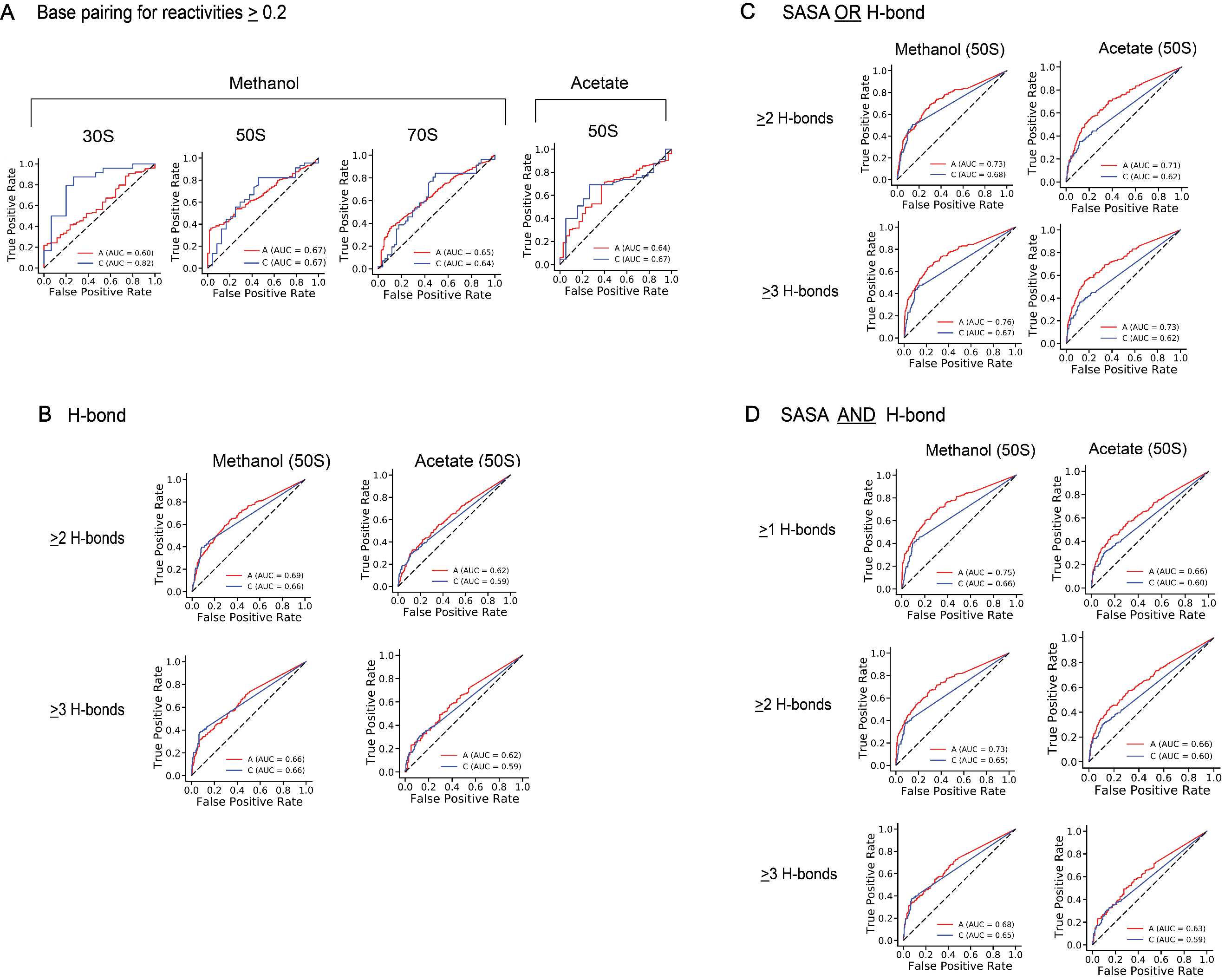
**

**Supplemental Fig. S9:** Additional ribosome structural features and *in vivo* DMS reactivity. (**A**) ROC curves that test binary of base pairing against DMS reactivities ≥0.2 in various ribosome subunits in methanol and acetate substrates. (**B**) ROC curves that test ≥ 2 and ≥ 3 hydrogen bonds at the WCF face (See Methods) for the 50S in methanol and acetate substrates. (**C**) ROC curves that combine SASA OR ≥ 2 or ≥ 3 hydrogen bonds on DMS reactive bases in 50S in methanol and acetate substrates. (**D**) ROC curves that combine SASA AND ≥ 1 as well as >2 and >3 hydrogen bond on DMS reactivity for the 50S in methanol and acetate substrates. SASA and distance cut-offs of < 1.4 Å^2^ and ≤ 3.5 Å, respectively, were applied.

**
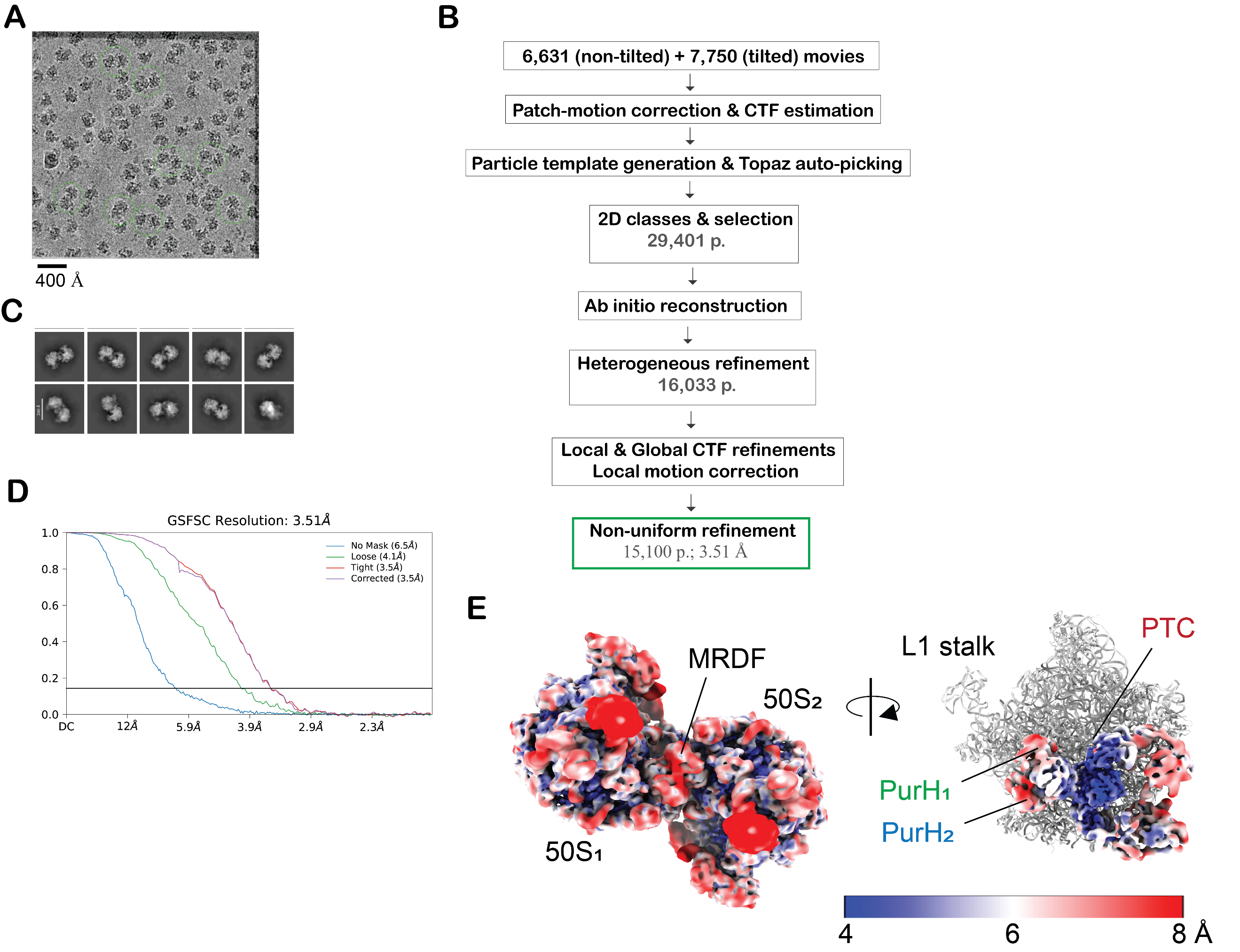
**

**Supplementary Fig. S10**: Cryo-EM data processing of the 50S subunit dimer with MRDF. (**A**) A representative micrograph showing particle distribution. (**B**) Cryo-EM data processing workflow. (**C**) Representative 2D class images. (**D**) Fourier shell correlation (FSC) plot for half-maps with 0.143 FSC criteria indicated nominal resolution at 3.51 Å. (**E**) Local resolution maps of 50S subunits dimer with MRDF (left) and MRDF (right).

**Supplementary Table S1:** Cryo-EM Data collection, refinement, and validation statistics

|  | Acetate-grown 50S | Methanol-grown 50S | Methanol-grown 70S | Methanol-grown 50S-50S dimer |
| --- | --- | --- | --- | --- |
| PDB | 9NRI | 9NTA | 9O17 | 9OU7 |
| EMBD | EMD-49734 | EMD-49757 | EMD-49998 | EMD-70864 |
| Data collection and processing | | | | |
| Magnification | 59,000 | 190,000 | 190,000 | |
| Voltage (kV) | 300 | 200 | 200 | |
| Electron dose (e^-^/Å^2^) | 50.79 | 50.00 | 50.00 | |
| Image pixel size (Å) | 1.1 | 0.733 | 0.733 | 0.977 |
| Defocus range (μm) | -0.8 to -2.0 | -0.6 to -2.0 | -0.6 to -2.0 | |
| Total number of images | 4,119 | 6,631 | 6,631 (non-tilted); 7,750 (tilted) | |
| Symmetry imposed | C1 | C1 | C1 | C2 |
| Final particle images (no.) | 61,932 | 197,922 | 91,385 | 15,100 |
| Refinement | | | | |
| Initial model used | 6SKF | 6SKF | 6SKF | 9NTA, Alphafold |
| Map resolution (Å)  FSC threshold 0.143 | 2.85 | 2.38 | 2.92 | 3.51 |
| Q-score | 0.70 | 0.77 | 0.63 | 0.33 |
| Model composition |  |  |  |  |
| Non-Hydrogen Atoms | 95,203 | 95,216 | 149,407 | 272,160 |
| Protein residues | 3,933 | 3,933 | 7,163 | 10,402 |
| Nucleotide residues | 3,012 | 3,012 | 4,366 | 6,024 |
| Ligands | Mg: 43; Zn:5 | Mg: 43; Zn:5; K: 11 | MG: 51; ZN:9 | - |
| R.m.s. deviations |  |  |  |  |
| Bond lengths (Å) | 0.006 | 0.005 | 0.004 | 0.007 |
| Bond angles (°) | 0.661 | 0.706 | 0.717 | 0.746 |
| ADP (B-factors) |  |  |  |  |
| Protein | 59.03 | 42.18 | 61.54 | 115.46 |
| Nucleotide | 62.58 | 40.47 | 66.20 | 129.55 |
| Ligand | 33.58 | 36.14 | 37.14 | - |
| Validation |  |  |  |  |
| MolProbity score | 1.87 | 1.63 | 2.14 | 2.29 |
| Clash score | 8.33 | 8.66 | 17.69 | 16.57 |
| Rotamer outliers (%) | 0.03 | 0.00 | 0.00 | 0.01 |
| Ramachandran plot |  |  |  |  |
| Favored (%) | 93.59 | 97.11 | 94.13 | 89.20 |
| Allowed (%) | 6.35 | 2.89 | 5.80 | 8.91 |
| Outliers (%) | 0.05 | 0.00 | 0.07 | 1.89 |

**Supplementary Table S2**: Analysis of ribosomal proteins among mesophilic and thermophilic species

|  | Euryarchaeota | | | | Thermoproteota | | | | Asgardarchaeota | Eukarya | |
| --- | --- | --- | --- | --- | --- | --- | --- | --- | --- | --- | --- |
|  | ***M. acetivorans*** | ***M. jannaschii*** | ***T. kodakarensis*** | ***P. furiosus*** | ***N. maritimus*** | ***N. viennensis*** | ***P. calidifontis*** | ***S. acidocaldarius*** | ***P. syntrophicum*** | ***S. cerevisiae*** | ***H. sapiens*** |
| eL8 | Yes | Yes | Yes | Yes | Yes | Yes | Yes | Yes | Yes | Yes | Yes |
| eL14 | -- | Yes | Yes | Yes | -- | -- | Yes | Yes | Yes | Yes | Yes |
| eL34 | -- | Yes | Yes | Yes | -- | -- | Yes | Yes | Yes | Yes | Yes |
| L41E | -- | Yes | Yes | Yes | -- | -- | -- | -- | -- | Yes | Yes |

Where “Yes” means gene (homolog) present

**Supplementary Table S3**: G+C content analysis (in percentages) of ribosomal RNAs among mesophilic and thermophilic species

|  | Mesophilic Bacteria | Thermophilic Bacteria | Mesophilic Archaea | | | Thermophilic Archaea | | | | | Eukarya | |
| --- | --- | --- | --- | --- | --- | --- | --- | --- | --- | --- | --- | --- |
| **sequence** | ***E. coli*** | ***T. thermophilus*** | ***N. maritimus*** | ***M. acetivorans*** | ***N. viennensis*** | ***M. jannaschii*** | ***T. kodakarensis*** | ***P. furiosus*** | ***P. calidifontis*** | ***S. acidocaldarius*** | ***H. sapiens*** |  |
| Whole genome | 50.8 | 69.0 | 34.2 | 42.7 | 52.7 | 31.4 | 52.0 | 40.8 | 57.2 | 36.7 | 41.6 |  |
| 16S/ 18S rRNA | 53.4 | 64.0 | 52.6 | 56.6 | 57.6 | 64.3 | 66.0 | 66.3 | 67.9 | 62.8 | 54.0 |  |
| 23S/ 28S rRNA | 53.4 | 63.6 | 50.1 | 53.0 | 56.0 | 63.3 | 65.2 | 66.0 | 67.9 | 60.4 | 62.4 |  |
| 5S rRNA | 63.3 | 67.2 | 54.2 | 57.1 | 62.1 | 68.3 | 72.2 | 71.4 | 71.6 | 64.8 | 60.0 |  |
| 5.8S rRNA |  |  |  |  |  |  |  |  |  |  | 57.7 |  |

*Escherichia coli (E. coli), Thermus thermophilus (T. thermophilus), Methanosarcina acetivorans (M. acetivorans), Methanocaldococcus jannaschii (M. jannaschii), Thermococcus kodakarensis (T. kodakarensis), Pyrococcus furiosus (P. furiosus), Nitrosopumilus maritimus (N. maritimus), Nitrososphaera viennensis (N. viennensis), Pyrobaculum calidifontis (P. calidifontis), Sulfolobus acidocaldarius (S. acidocaldarius), Promethearchaeum syntrophicum (P. syntrophicum), Saccharomyces cerevisiae (S. cerevisiae), Homo sapiens (H. sapiens)*

**Supplementary Table S4:** Structural factors of differentially DMS-reactive nucleotides.

| **Nucleotide position ^a^Value(Value)** | **Nucleotide** | **Reactivity (Methanol)** | **Reactivity (Acetate)** | **Reactivity Difference (Methanol - Acetate)** | **Canonically base paired** | **SASA Å^2^ (Methanol)** | **SASA Å^2^ (Acetate)** | **SASA Å^2^ (Methanol - Acetate)** | **Number of H-bonding (Methanol)** | **Number of H-bonding (Acetate)** | **Number of H-bonding (Methanol - Acetate)** | **Protein-RNA Contacts (Methanol)** | **Protein-RNA Contacts (Acetate)** | **Protein Contacts Difference (Methanol - Acetate)** | **RMSD Å  (\|Methanol-Acetate\|)** |
| --- | --- | --- | --- | --- | --- | --- | --- | --- | --- | --- | --- | --- | --- | --- | --- |
| **2853 (2858)** | A | 1.17 | 0.18 | 0.99 | no | 10.66 | 10.66 | 0.00 | 2 | 2 | 0 | 0 | 0 | 0 | 2.57 |
| **1573 (1578)** | A | 0.92 | 0.00 | 0.92 | no | 20.23 | 21.32 | -1.09 | 0 | 2 | -2 | 0 | 0 | 0 | 2.05 |
| **1910 (1915)** | A | 1.17 | 0.33 | 0.83 | no | 0.00 | 0.00 | 0.00 | 8 | 6 | 2 | 0 | 0 | 0 | 1.93 |
| **1282 (1287)** | A | 0.79 | 0.00 | 0.79 | A-U | 0.00 | 0.00 | 0.00 | 2 | 2 | 0 | 0 | 0 | 0 | 1.25 |
| **1940 (1945)** | A | 0.74 | 0.00 | 0.74 | no | 9.30 | 20.50 | -11.21 | 1 | 1 | 0 | 0 | 0 | 0 | 1.84 |
| **1890 (1895)** | A | 0.95 | 0.21 | 0.74 | no | 0.00 | 0.00 | 0.00 | 3 | 3 | 0 | 0 | 0 | 0 | 1.84 |
| **1781 (1786)** | A | 0.70 | 0.03 | 0.66 | A-U | 0.00 | 0.00 | 0.00 | 5 | 4 | 1 | 10 | 4 | 6 | 1.84 |
| **1963 (1968)** | C | 1.35 | 0.70 | 0.65 | no | 1.37 | 1.09 | 0.27 | 2 | 3 | -1 | 0 | 0 | 0 | 1.84 |
| **1563 (1568)** | C | 0.89 | 0.25 | 0.65 | no | 0.82 | 1.91 | -1.09 | 1 | 1 | 0 | 0 | 0 | 0 | 1.84 |
| **2842 (2847)** | A | 0.93 | 0.30 | 0.63 | no | 7.93 | 10.66 | -2.73 | 1 | 1 | 0 | 0 | 0 | 0 | 1.84 |
| **2147 (2152)** | A | 0.65 | 0.03 | 0.62 | no | 0.00 | 0.00 | 0.00 | 3 | 7 | -4 | 0 | 0 | 0 | 1.84 |
| **804 (809)** | A | 0.65 | 0.07 | 0.58 | A-U | 0.00 | 0.00 | 0.00 | 3 | 3 | 0 | 14 | 5 | 9 | 1.84 |
| **1892 (1897)** | C | 0.58 | 0.00 | 0.58 | no | 0.27 | 0.82 | -0.55 | 1 | 1 | 0 | 0 | 0 | 0 | 1.84 |
| **799 (804)** | A | 1.43 | 0.86 | 0.57 | no | 11.76 | 14.76 | -3.01 | 2 | 0 | 2 | 0 | 0 | 0 | 1.84 |
| **1442 (1447)** | A | 0.56 | 0.00 | 0.56 | no | 0.00 | 0.00 | 0.00 | 7 | 6 | 1 | 5 | 4 | 1 | 1.84 |
| **985 (990)** | C | 0.71 | 0.17 | 0.54 | no | 0.00 | 0.00 | 0.00 | 2 | 2 | 0 | 0 | 0 | 0 | 1.84 |
| **1315 (1320)** | A | 0.56 | 0.00 | 0.56 | A-U | 0.00 | 0.00 | 0.00 | 6 | 5 | 1 | 5 | 4 | 1 | 1.84 |
| **1839 (1844)** | A | 0.59 | 0.08 | 0.52 | no | 0.00 | 1.09 | -1.09 | 4 | 2 | 2 | 1 | 0 | 1 | 1.84 |
| **977 (982)** | A | 1.80 | 1.28 | 0.51 | no | 21.32 | 21.32 | 0.00 | 0 | 0 | 0 | 0 | 0 | 0 | 1.84 |
| **2263 (2268)** | C | 0.51 | 0.00 | 0.51 | no | 5.74 | 5.47 | 0.27 | 0 | 0 | 0 | 2 | 4 | -2 | 1.84 |
| **1589 (1594)** | A | 1.56 | 1.05 | 0.51 | no | 9.02 | 9.30 | -0.27 | 3 | 3 | 0 | 0 | 0 | 0 | 1.84 |
| **1474 (1479)** | A | 0.50 | 0.00 | 0.50 | no | 4.10 | 6.83 | -2.73 | 5 | 3 | 2 | 0 | 0 | 0 | 1.84 |
| **868 (873)** | A | 0.35 | 1.36 | -1.02 | no | 1.91 | 3.01 | -1.09 | 7 | 5 | 2 | 1 | 0 | 1 | 0.81 |
| **166 (171)** | C | 0.00 | 0.80 | -0.80 | A-U | 0.00 | 0.00 | 0.00 | 7 | 5 | 2 | 6 | 4 | 2 | 1.13 |
| **2808 (2813)** | C | 0.00 | 0.73 | -0.73 | G-C | 0.00 | 0.00 | 0.00 | 7 | 5 | 2 | 4 | 3 | 1 | 1.84 |
| **2609 (2614)** | A | 0.17 | 0.80 | -0.63 | no | 0.00 | 0.00 | 0.00 | 7 | 7 | 0 | 9 | 7 | 2 | 1.84 |
| **1529 (1534)** | C | 0.00 | 0.63 | -0.63 | G-C | 0.00 | 0.00 | 0.00 | 3 | 3 | 0 | 5 | 5 | 0 | 1.84 |
| **2780 (2785)** | A | 0.00 | 0.63 | -0.63 | no | 3.83 | 4.65 | -0.82 | 1 | 0 | 1 | 10 | 6 | 4 | 1.84 |
| **1672 (1677)** | C | 0.80 | 1.38 | -0.59 | no | 3.28 | 4.10 | -0.82 | 3 | 4 | -1 | 3 | 3 | 0 | 1.84 |
| **867 (872)** | A | 0.16 | 0.71 | -0.55 | no | 0.00 | 2.46 | -2.46 | 2 | 2 | 0 | 14 | 12 | 2 | 1.84 |
| **1562 (1567)** | A | 0.53 | 1.06 | -0.53 | no | 8.20 | 9.57 | -1.37 | 4 | 3 | 1 | 0 | 0 | 0 | 1.84 |
| **588 (593)** | A | 1.90 | 2.42 | -0.53 | no | 6.56 | 5.47 | 1.09 | 6 | 4 | 2 | 0 | 0 | 0 | 1.84 |
| **1341 (1346)** | A | 0.11 | 0.63 | -0.52 | no | 0.00 | 0.00 | 0.00 | 6 | 4 | 2 | 0 | 0 | 0 | 1.84 |
| **378 (383)** | C | 0.53 | 1.03 | -0.50 | no | 0.82 | 0.82 | 0.00 | 1 | 0 | 1 | 0 | 0 | 0 | 1.84 |

The table lists the 34 differentially reacting nucleotides with the methanol and acetate reactivity difference (|methanol-acetate| > 0.5) reported in the DMS-probing in *M. acetivorans* publication (1). ^a^Value outside of the parentheses is from the present study. Value inside of parentheses is from our previous study (1). For these bases, the structural factors tabulated are canonical base pairing state, solvent accessible surface area (SASA) of the DMS-reactive nitrogen, number of hydrogen bonding interactions, number of protein-RNA contacts, and the RMSD of the nucleotide. The top part of the table are bases with reactivity higher in methanol (teal) and the bottom part indicates bases with reactivity higher in acetate (orange). There are columns showing differences between SASA, differences in number of hydrogen bonding interactions, and differences in numbers of protein-RNA contacts. No strong trends were observed for these factors and differential DMS reactivity.

**Supplementary Table S5:** ROC Curves comparing structural features with DMS reactivity.

|  | **Methanol (AUC)** | | **Acetate (AUC)** | |
| --- | --- | --- | --- | --- |
| **Binary** | **A** | **C** | **A** | **C** |
| Paired 30S (16S rRNA) | 0.58 | 0.56 |  |  |
| Paired 30S DMS Reactivity > 0.2 | 0.60 | 0.82 |  |  |
| Paired 50S (23S rRNA + 5S rRNA) | 0.59 | 0.64 | 0.60 | 0.59 |
| Paired 50S DMS Reactivity > 0.2 | 0.67 | 0.67 | 0.64 | 0.67 |
| Paired 70S (23S rRNA + 5S rRNA + 16S rRNA) | 0.60 | 0.63 |  |  |
| Paired 70S DMS Reactivity > 0.2 | 0.65 | 0.64 |  |  |
| 30S SASA <1.4Å^2^ | 0.71 | 0.62 |  |  |
| 50S SASA <1.4Å^2^ | 0.76 | 0.66 | 0.72 | 0.62 |
| 70S SASA < 1.4Å^2^ | 0.67 | 0.61 |  |  |
| > 1-Hbond 30S | 0.66 | 0.55 |  |  |
| > 1-Hbond 50S | 0.73 | 0.70 | 0.65 | 0.60 |
| > 2-Hbond 50S | 0.69 | 0.66 | 0.62 | 0.59 |
| > 3-Hbond 50S | 0.66 | 0.66 | 0.62 | 0.59 |
| > 1-Hbond 70S | 0.71 | 0.65 |  |  |
| > 1-Hbond 50S OR SASA <1.4Å^2^ | 0.77 | 0.73 | 0.73 | 0.64 |
| > 2-Hbond 50S OR SASA <1.4Å^2^ | 0.73 | 0.68 | 0.71 | 0.62 |
| > 3-Hbond 50S OR SASA <1.4Å^2^ | 0.76 | 0.67 | 0.73 | 0.62 |
| > 1-Hbond 50S AND SASA <1.4Å^2^ | 0.75 | 0.66 | 0.66 | 0.60 |
| > 2-Hbond 50S AND SASA <1.4Å^2^ | 0.73 | 0.65 | 0.66 | 0.60 |
| > 3-Hbond 50S AND SASA <1.4Å^2^ | 0.68 | 0.65 | 0.63 | 0.59 |

This table captures a vast number of the area under the curves (AUC) for A and C against various structural factors and DMS thresholds shown in the “Binaries” column. The factors include canonical base pairing, canonical base pairing considering benchmarks of DMS reactivities > 0.2, SASA, and numbers of hydrogen bonding interactions near/at the WCF atoms. Hydrogen bonding was also combined with SASA. For the most part, SASA increased the AUC for most independent factors. 30S (SSU), 50S (LSU), and (70S) plots were only made for methanol whereas only the 50S curves were made for acetate.

**REFERENCES**

1. Williams, A. M., Jolley, E. A., Santiago-Martínez, M. G., Chan, C. X., Gutell, R. R., Ferry, J. G., et al. (2023) *In vivo* structure probing of RNA in Archaea: novel insights into the ribosome structure of *Methanosarcina acetivorans*. *RNA* **29**.
