# Supplementary material for: Cryo-EM study and in vivo chemical mapping of the Methanosarcina acetivorans ribosome and its dimerization via a repurposed enzyme and translation factor
Source: J Biol Chem. 2025 Sep 4;301(11):110686. doi: 10.1016/j.jbc.2025.110686 (PMC12605026; doi:10.1016/j.jbc.2025.110686)
Supplement: Movie legend [file mmc2.docx]

**SUPPLEMENTAL INFORMATION**

SMovie 1. Cryo-EM density map of the *M. acetivorans* 70S ribosome. Related to Fig. 1A.

SMovie 2. Structure of the *M. acetivorans* 70S ribosome. Related to Fig. 1B.

SMovie 3. Cryo-EM density map of the *M. acetivorans* ribosome 50S subunit dimer with MRDF. Related to Fig. 6A.

SMovie 4. Structure of the *M. acetivorans* ribosome 50S subunit with MRDF. Related to Fig. 6B.
